# Textiles impregnated with antimicrobial substances in healthcare services: systematic review

**DOI:** 10.3389/fpubh.2023.1130829

**Published:** 2023-05-11

**Authors:** Guilherme Schneider, Leticia Genova Vieira, Herica Emilia Félix de Carvalho, Álvaro Francisco Lopes de Sousa, Evandro Watanabe, Denise de Andrade, Renata Cristina de Campos Pereira Silveira

**Affiliations:** ^1^Ribeirão Preto College of Nursing, University of São Paulo, Ribeirão Preto, Brazil; ^2^Global Health and Tropical Medicine, Institute of Hygiene and Tropical Medicine, New University of Lisbon, Lisbon, Portugal; ^3^Institute of Education and Research, Hospital Sírio Libanês, São Paulo, Brazil; ^4^School of Dentistry of Ribeirão Preto, University of São Paulo, Ribeirão Preto, Brazil

**Keywords:** textiles, antimicrobial agents, health services, biosafety, infection control

## Abstract

**Background:**

Antimicrobial textiles have proved to be a promising biosafety strategy. Thus, the current study was focused on identifying which antimicrobial substances impregnated in textiles used in healthcare services confer efficacy in reducing the microbial load present in these textiles and/or the Healthcare-Associated Infection (HAI) rates, when compared to conventional textiles.

**Methods:**

A systematic review of intervention studies using MEDLINE *via* the PubMed portal, EMBASE, CINAHL, Web of Science, Scopus, Google Scholar and medRxiv. The studies identified were selected according to eligibility criteria and submitted to data extraction and methodological quality evaluation through Joanna Briggs Institute specific tools. The outcomes were synthesized qualitatively.

**Results:**

23 studies were selected to comprise the final sample, in which antimicrobial textiles were used by hospitalized patients, by health professionals during work shifts and in inanimate healthcare environments.

**Conclusions:**

Copper, silver, zinc oxide, titanium and silver-doped titanium impregnated in textiles used by patients confer efficacy in reducing the microbial load of these textiles and/or the HAI rates. Quaternary ammonium, chlorhexidine, silver and copper together, quaternary ammonium, alcohols and isothiazolone derivatives together, chitosan and dimethylol dimethyl hydantoin together, all impregnated in textiles used by health professionals confer efficacy in reducing the microbial load of these textiles. Quaternary ammonium impregnated in textiles used in inanimate healthcare environments confers efficacy in reducing the microbial load of these textiles.

## Highlights

- Textiles impregnated with antimicrobial substances can be used in health services.
- Antimicrobial textiles provide biosafety to patients and health professionals.
- Compared to conventional textiles, antimicrobial textiles have lower microbial load.
- Antimicrobial textiles can be a viable alternative to reduce HAIs.

## Background

The patient's surroundings can be considered as sources of microbial contamination due to factors such as high frequency of touch and mutual contact by health professionals during care activities, as well as by the patients themselves and their visitors, favoring cross-contamination (1). In this context, the textile materials found in healthcare services, whether in inanimate environments in general, in the professionals' uniforms or in clothing and bed linen used by the patients during the hospitalization regime, are not exempt from microbial contamination, proliferation and dissemination (2).

For example, the scientific literature points to the wide microbial contamination of privacy curtains in clinical settings, (3, 4) as well as of the health professionals' white coats, (5, 6) in addition to the clothing and bed linen used by the patients during the hospitalization period (7, 8). There are also possible indications for relationships of microbial contamination in different textile materials used by the patients and health professionals and in the inanimate healthcare environments, with occurrence of Healthcare-Associated Infections (HAIs) and infectious outbreaks in hospital services (9, 10).

Thus, there is an interest in textile impregnation, in order to provide those materials with antimicrobial properties that tend to minimize contamination and microbial load, thus reducing the biological risks. This process can be basically performed through two different methods, namely: previous incorporation of the antimicrobial agent into the textile fiber matrix, into the spinning process; or coating, from specific techniques that promote adhesion of the antimicrobial agent to the textile substrate during the finishing process (11). The substances with antimicrobial properties used in impregnation of textiles can be both organic (such as quaternary ammonium compounds, halamines, polybiguanides, triclosan, chitosan and bioactive plant-based compounds) and inorganic (such as nanoparticles and metal oxides) (12).

Despite being a promising strategy, wide implementation of these antimicrobial textiles in healthcare services should be, above all, cautious, as the scientific literature on the theme points to important contradictions in laboratory studies regarding the results in relation to microbial load reduction, including drug-resistant microorganisms, (13, 14) as well as cytotoxicity (15, 16). In addition to that, in real healthcare conditions, clinical studies with different use configurations of textiles impregnated with antimicrobials employed by professionals or hospitalized patients collectively obtained intriguing results, being considered unsatisfactory regarding reduction of the microbial load on health professionals' clothes, but satisfactory in relation to the reduction of HAIs, respectively (17).

Furthermore, the potential for induction of microbial resistance to the substances impregnated in these textiles should be considered, mainly when the antimicrobial and/or impregnation method selected favor controlled release mechanisms and, consequently, considerable leaching in a wet medium. This gradual and persistent release is followed by a reduction of the antimicrobial concentration in the textile at levels below the Minimum Inhibitory Concentration (MIC), that is, the antimicrobial efficacy limit that can induce development of microbial resistance (12). It should also be considered that there are yet no definitive answers as to the potential of certain substances impregnated in textiles to promote selection of microorganisms resistant to other antimicrobials, even pharmacological ones (18), which increases the risk for the advent of cross-resistance, co-resistance and resistance by co-regulation to antimicrobials (19).

In this context, the current study focused on identifying which antimicrobial substances impregnated in textiles used in healthcare services confer efficacy in reducing the microbial load present in these textiles and/or the HAI rates, when compared to conventional textiles.

## Methods

### Study design

This study is a systematic review of the scientific literature, focused on the research studies where a given intervention was implemented in healthcare services, as well as evaluation of its effectiveness (20) reported in accordance with the guidelines proposed by the Preferred Reporting Items for Systematic Reviews and Meta-Analysis (PRISMA) (21).

### Systematic review protocol

The systematic review protocol was registered in the Open Science Framework (OSF) platform, under DOI 10.17605/OSF.IO/M685U on October 21, 2021, and can be consulted in full *via* the following access link: https://osf.io/m685u.

### Research question

The research question was prepared with the help of the PICO strategy, so that:

- (P)roblem: microbial load present in the textiles used by patients and health professionals and in the inanimate healthcare environments, and HAI rates;
- (I)ntervention: textiles impregnated with antimicrobial substances;
- (C)omparison: conventional textiles (devoid of any type of impregnation);
- (O)utcome: reduction of the microbial load present in the textiles used by patients and health professionals and in inanimate healthcare environments, and/or reduction of the HAI rates.

Thus, the following research question was defined: “Which are the antimicrobial substances impregnated in textiles used by patients and health professionals and in inanimate healthcare environments, which confer efficacy in reducing the microbial load present in these textiles and/or the HAI rates, when compared to conventional textiles?”.

### Additional outcomes

The adverse events (cutaneous signs and symptoms) presented by the patients and/or health professionals after using textiles impregnated with antimicrobial substances, according to each type of textile material and antimicrobial substance, were considered as additional outcomes to be determined and synthesized in this review.

### Eligibility criteria

Among the eligibility criteria, the following inclusion criteria were considered: primary studies with an intervention design (clinical trials and quasi-experimental studies) that addressed the use of textiles impregnated with antimicrobial substances in healthcare services, quantitatively evaluating variation of the microbial load present in these textiles and/or of the HAI rates (by means of a theoretical framework and/or indicators), according to their use.

In general, the studies on this theme developed under real healthcare conditions do not present details of how the method to manufacture/impregnate the textiles with antimicrobials took place. As a result, it was agreed to adopt as eligible those studies in which the health institutions themselves replaced their conventional textiles (devoid of any type of impregnation) by impregnated textiles/antimicrobials, as well as those where the researchers reported the supply of impregnated/antimicrobial textiles for the health services. In addition, in view of the objectives proposed, it is also noted that the only studies that were considered eligible were those that specified the antimicrobial substances (or at least one of those substances) impregnated in the textiles, as well as their applicability with regard to using them in inanimate healthcare environments, whether by health professionals during care activities and/or by patients during the hospitalization period.

As for the exclusion criteria, the studies conducted under the following use configurations of the antimicrobial-impregnated textiles were considered ineligible:

- Use by patients during the hospitalization period: impregnated/antimicrobial textiles for personal hygiene care (such as cloths impregnated for antisepsis and bathing, as well as impregnated diapers and tampons), dressings (such as impregnated gauze for wound covering), or adjuvant treatment of skin tissue disorders such as atopic dermatitis;
- Use by health professionals during their respective work shifts: impregnated/antimicrobial textiles such as Personal Protective Equipment (PPE) directed to the precaution regarding droplets or aerosols (such as impregnated facial protection masks), or for specific healthcare interventions (such as aprons and impregnated incision fields for use in surgical procedures);
- Use in inanimate healthcare environment: impregnated/ antimicrobial textiles for performing environmental hygiene procedures (such as impregnated cloths for disinfection of surfaces).

In addition, once again based on the objectives proposed, the studies considered eligible were those where concomitant use of other antimicrobial surfaces took place, in addition to the intervention of interest (impregnated/antimicrobial textiles) in the health service; as well as those that do not present sufficient information/data for characterization and analysis of the methodological quality/risk of bias. Finally, materials published on the theme such as editorials, letters to the editor, books, book chapters, theses, dissertations and abstracts presented in scientific events were considered ineligible.

### Process to identify studies in the scientific literature

The information sources consulted were the following databases: Medical Literature Analysis and Retrieval System Online (MEDLINE) *via* the PubMed portal, Excerpta Medica DataBase (EMBASE), Cumulative Index to Nursing and Allied Health Literature (CINAHL) *via* the EBSCOhost platform, Web of Science and Scopus. In addition, the Gray Literature was explored through Google Scholar (which also allows retrieving studies indexed in the databases searched), as well as the medRxiv preprint database.

The search strategy was formulated through the combination of controlled descriptors and keywords related to the theme of interest, being adapted to each of the aforementioned information sources, that is, respecting their particularities. It is noteworthy that, in order to identify the studies referring to the research question in the most comprehensive possible way, no filters related to publication period and language were used.

In the exceptional case of Google Scholar, as it is a search engine that tends to identify endless results, it was decided to delimit the process corresponding to analysis and selection of studies that met the eligibility criteria only to the first 100 results identified in order of relevance. Thus, so as to avoid potential losses in the identification of eligible studies resulting from this process, four different search strategies were structured and applied in this search platform (Supplementary material 1).

Additionally, a manual search was conducted for other studies that met the previously established eligibility criteria, analyzing the lists of references cited by the studies identified in the databases and in the Gray Literature, which were considered eligible in the analysis and selection process.

### Analysis and selection process corresponding to the studies identified in the scientific literature

Initially, all 2,026 records identified in the databases and in the Gray Literature were imported into the EndNote Basic^®^ software (Clarivate Analytics), a reference manager where 653 duplicates were removed, totaling 1,373 records screened that were subsequently imported into the Rayyan^®^ software (Qatar Computing Research Institute), where the process to analyze and select the studies based on the eligibility criteria was conducted.

Independently and blindly, two researchers conducted the process for the analysis and selection of the studies, in two phases: in phase 1, after reading the titles and abstracts of all 1,373 records screened, 1,322 were excluded for not responding to the review objectives. Consequently, 51 reports progressed to Phase 2 where, after full-text reading, 29 were excluded due to the following reasons:

- Reason 1: Material on the theme published, such as editorial, letter to the editor, book, book chapter, thesis, dissertation or abstract presented in a scientific event, which resulted in the exclusion of 16 reports;
- Reason 2: The study does not specify the antimicrobial substance impregnated in the textiles, which resulted in the exclusion of 3 reports;
- Reason 3: The study does not specify applicability of the antimicrobial textiles in the health service, which resulted in the exclusion of 2 reports;
- Reason 4: The study does not assess microbial load in the antimicrobial textiles and/or the HAI rates by means of a theoretical framework or indicators, which resulted in the exclusion of 1 report;
- Reason 5: In addition to the intervention of interest (antimicrobial textiles), the study addresses concomitant use of other antimicrobial surfaces in the health service, which resulted in the exclusion of 6 reports;
- Reason 6: The study does not present sufficient information/data for characterization and analysis of methodological quality/risk of bias, which resulted in the exclusion of 1 report.

Thus, 22 studies were considered eligible for inclusion in this review.

Complementing this sample, the lists of references cited by all 22 studies were examined, which resulted in the identification of another 3 potentially eligible records (after reading titles and abstracts), from which 2 reports were excluded (after full-text reading) due to the following reasons:

- Reason 5: In addition to the intervention of interest (antimicrobial textiles), the study addresses concomitant use of other antimicrobial surfaces in the health service, which resulted in the exclusion of 1 reports;
- Reason 7: The study investigates a given non-textile antimicrobial material fixed to conventional textiles (non-antimicrobial), which resulted in the exclusion of 1 report.

**Figure 1:**
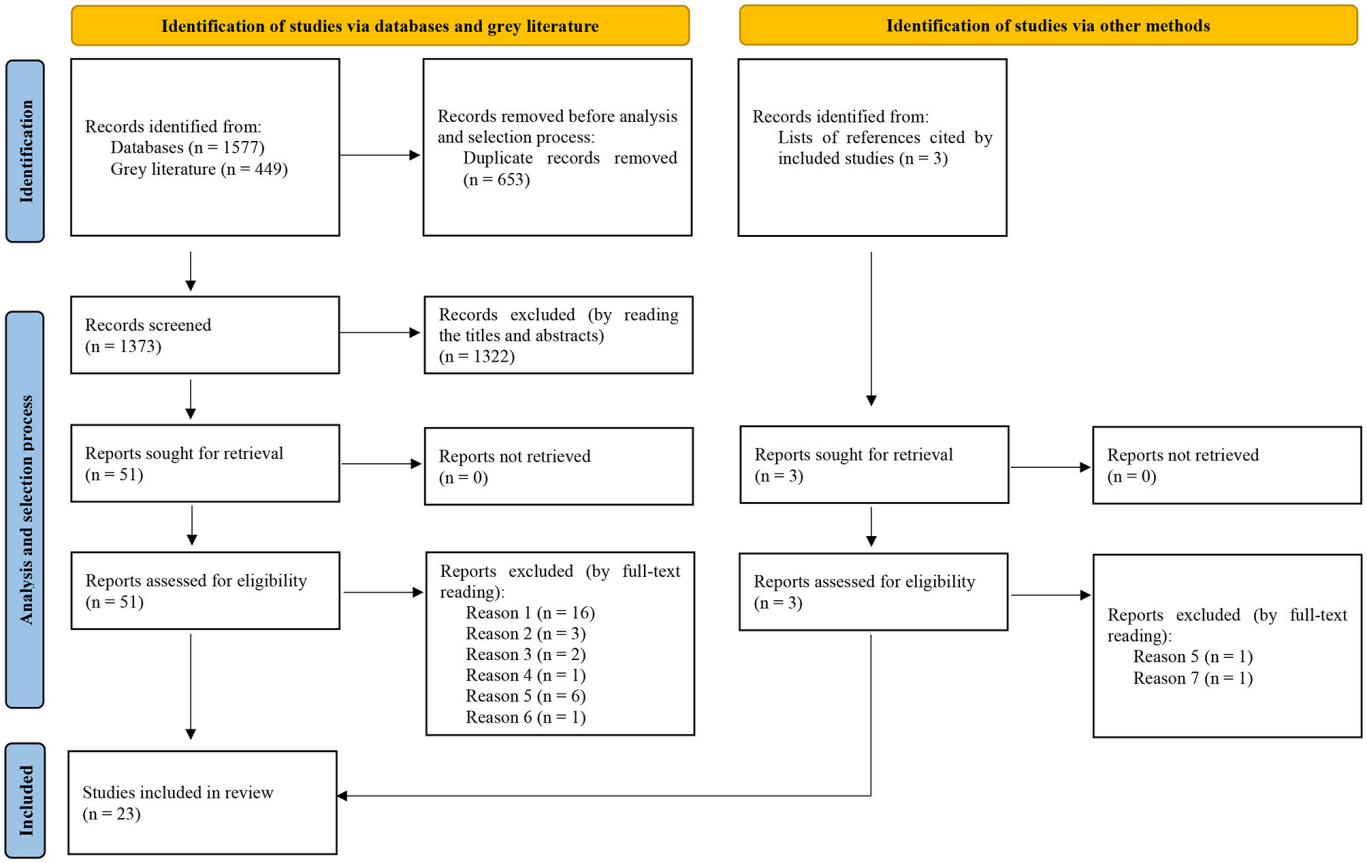
Flowchart, adapted from PRISMA, corresponding to the process of analysis and selection of studies identified in the scientific literature.

Thus, only 1 study was considered eligible through this process, resulting in a final sample of 23 studies included in this knowledge synthesis. This process can be visualized with more details in Figure 1 below.

Any and all possible disagreements in this process of analysis and selection of studies were resolved by a third researcher with expertise in the theme of interest.

The reports excluded (after full-text reading) were referenced and presented together with their respective reasons for exclusion in Supplementary material 2.

### Process to extract data from the studies selected

Once again independently and blindly, two researchers conducted the process for extracting the following data from the studies selected:

- Characteristics of the study: design, sample size, locus/country in which the study was developed and follow-up period;
- Characteristics of the intervention: types of textiles, antimicrobial substances impregnated in the textiles, applicability of these textiles in health services, and hygiene settings of these textiles;
- Assessment method of the microbial load in the textiles, HAI rates and adverse events;
- Results of microbial load present in the textiles, HAI rates and adverse events.

Due to the different and extensive microbiological assessments performed in the studies selected, it was decided to prioritize the extraction of non-specific microbial load data in the conventional and impregnated/antimicrobial textiles before and after their use, as presented by the studies. In the studies that did not present these data, it was agreed to prioritize extraction of the specific microbial load data or microbial load in each sampled area of the textiles before and after their use, as presented.

Regarding assessment of the HAI rates, it was agreed to prioritize extraction of the non-specific HAI data and indicators before and after using conventional and impregnated/antimicrobial textiles, as presented by the studies. In the studies that did not present these data, it was agreed to prioritize extraction of the specific HAI data in relation to the etiological agents before and after using conventional and impregnated/antimicrobial textiles, as presented.

After completing this process, both researchers cross-checked the data retrieved, and any and all divergences were resolved by discussion and mutual agreement. In the event of any disagreement, a third researcher with expertise in the theme of interest was available for consultation and final decision-making.

Subsequently, the data retrieved referring to each of the studies selected were recorded in study characterization charts, namely:

- Characterization chart of studies in which the textiles impregnated with antimicrobials were used by patients during the hospitalization period;
- Characterization chart of studies in which the textiles impregnated with antimicrobials were used by health professionals during their respective work shifts;
- Characterization chart of studies in which the textiles impregnated with antimicrobials were used in inanimate healthcare environments.

### Methodological quality assessment (risk of bias) corresponding to the studies selected

The methodological quality assessment (risk of bias) corresponding to each of the studies selected was performed through specific and appropriate Critical Appraisal Tools for each study design, made available by the Joanna Briggs Institute (JBI) (20).

These tools consist of different topics, which are filled out with the “Yes”, “No”, “Unclear” or “Not applicable” answers, according to the diverse information presented by the studies. Classification of the methodological quality of the studies is based on the percentage of “Yes” answers obtained for the topics that comprise the tool used, and it is the researchers themselves who previously define how the scoring system (cutoff points/percentages) will be constituted to classify methodological quality (20).

In this review, it was defined that, regardless of the tool, the topics that obtained the “Not applicable” answer would not be considered for calculation of the percentage of “Yes” answers, and that the methodological quality of the studies would be classified according to the following scoring system:

- Low methodological quality (high risk of bias): if the study evaluated reaches <50% of “Yes” answers to the topics of the tool used;
- Moderate methodological quality (moderate risk of bias): if the study evaluated reaches 50% to 74% of “Yes” answers to the topics of the tool used;
- High methodological quality (low risk of bias): if the study evaluated reaches 75% or more “Yes” answers to the topics of the tool used.

This process to assess the methodological quality of the studies selected was also carried out by two researchers, independently and blindly, and a third researcher with expertise in these tools was called upon to resolve any and all divergences.

### Synthesis of the results

The synthesis of the results was presented qualitatively, describing in general the data referring to the microbial load present in the textiles, the HAI rates and the adverse events presented by the patients and health professionals, according to the antimicrobial substances impregnated in the textiles used and their applicability in healthcare services, also considering the methodological quality of the studies selected.

It was not possible to perform a quantitative (statistical) synthesis of the results due to the marked heterogeneity of methodological configurations across the studies selected, as well as to their methodological quality.

### Assessment of the certainty of the synthesized evidence

Due to the impossibility of performing a quantitative (statistical) synthesis of the results, it was decided not to assess the certainty of the synthesized evidence (qualitatively) through the Grading of Recommendations Assessment, Development and Evaluation (GRADE) system (22), as previously planned in the systematic review protocol.

## Results

The 23 studies selected to comprise this systematic review, in which the textiles impregnated with antimicrobial substances were used by patients during the hospitalization period, by health professionals during their respective work shifts, and in inanimate healthcare environments, were characterized in Charts 1–3, respectively.

**Chart 1:** Characterization of studies selected in which the textiles impregnated with antimicrobial substances were used by patients during the hospitalization period.

**Identification of the study**	**Characteristics of the study**		**Characteristics of the experiment**			**Assessment method**	**Results**
	**Design and sample size**	**Locus/Country and follow-up period**	**Types of textiles and antimicrobial substances**	**Applicability in health service**	**Hygiene settings of the textiles**		
Marik et al. (23)	Controlled, randomized and cross-over clinical trial^*^. Control group: 645 patients, 2,141 patients-days; Intervention group: 637 patients, 2,185 patients-days.	General ICU of the Sentara Norfolk General Hospital, USA. Follow-up period: from January to December 2014 (two consecutive 23-week periods, separated by two washout weeks).	Control group: unimpregnated (not specified) textiles; Intervention group: textiles (not specified) impregnated with copper oxide.	Sheets, pillowcases, bath and face towels, and clothing (aprons) used by patients during the hospitalization period.	Control and intervention groups: the unimpregnated and impregnated textiles were subjected to the washing process separately, but in the same way (not specified).	Assessment of HAI rates based on a theoretical framework.^a^	Assessment of HAI rates: in the control and intervention groups, respectively: 28 (4.3%) (13 per 1,000 patients-days) and 25 (3.9%) (11.4 per 1,000 patients-days) (*p =* 0.6).
Balachandran et al. (24)	Quasi-experimental study. Sample sizes not reported.	Five community hospitals (country not specified). Control period: from July 2013 to December 2015; Intervention period: from January 2016 to June 2018 (the intervention was performed in three of the five hospitals during the first 18 months, and in all five hospitals in the last 12 months).	Control period: unimpregnated (not specified) textiles; Intervention period: textiles (not specified) impregnated with silver ions.	Sheets, pillowcases and clothing (aprons) used by patients during the hospitalization period.	Control period: textiles subjected to the usual washing process (not specified); Intervention period: textiles subjected to the usual washing process (not specified), with automated addition of an ionic silver solution, during the rinse cycle.	Assessment of HAI rates based on a theoretical framework (not fully specified).	Assessment of HAI rates: in general, in the three hospitals that only received impregnated textiles during the 30 months of the intervention period, there was a 42% reduction in the HAI rates, when compared to the control period (*p < * 0.0001).
Madden et al. (25)	Quasi-experimental study. Control period: 29,342 patients-days; Intervention period: 25,243 patients-days.	Long-term acute care hospital located in Charlottesville, USA. Control period: from July 2012 to September 2014; Intervention period: from October 2014 to December 2016; Control period (additional): from January 2017 to October 2017.	Control periods: unimpregnated (not specified) textiles; Intervention period: textiles (not specified) impregnated with copper.	Sheets, pillowcases, bath and face towels used by patients during the hospitalization period.	Control period: not specified; Intervention period: textiles subjected to the washing process, in accordance with the manufacturer's recommendations (not specified).	Assessment of HAI rates based on a theoretical framework.^b^	Assessment of HAI rates: the following was identified in the control and intervention periods, respectively: 44 (1.5 per 1,000 patients-days) and 70 (2.8 per 1,000 patients-days) infection events caused by *Clostridium difficile* (*p =* 0.023); and 9 (0.3 per 1,000 patients-days) and 11 (0.4 per 1,000 patients-days) infection events caused by multidrug-resistant microorganisms (*p =* 0.313).
Butler (26)	Quasi-experimental study. Pre-intervention periods A1, A2 and A3: 29,865, 59,662 and 81,448 patients-days, respectively; Post-intervention periods B1, B2 and B3: 34,625, 70,326 and 94,125 patients-days, respectively.	Six small- to medium-sized hospitals in the Sentara Health System, USA. Pre-intervention periods A1, A2 and A3: from May to July 2016; from May to October 2016; and from May to December 2016, respectively; Post-intervention periods B1, B2 and B3: from May to July 2017; from May to October 2017; and from May to December 2017, respectively.	Pre-intervention periods A1, A2 and A3: unimpregnated (not specified) textiles; Post-intervention periods B1, B2 and B3: textiles (not specified) impregnated with copper oxide.	Sheets, pillowcases, blankets, bath and face towels, and clothing (aprons) used by patients during the hospitalization period.	Not specified.	Assessment of HAI rates based on a theoretical framework (not fully specified).	Assessment of HAI rates: in periods A1, A2 and A3, 25 (0.84 per 10,000 patients-days), 48 (0.80 per 10,000 patients-days) and 62 (0.76 per 10,000 patients-days) infections by *Clostridium difficile* and multidrug-resistant microorganisms were identified, respectively, when compared to periods B1, B2 and B3, where 12 (0.34 per 10,000 patients-days) [59.8% reduction (*p < * 0.01)], 34 (0.48 per 10,000 patients-days) [39.9% reduction (*p < * 0.05)] and 45 (0.48 per 10,000 patients-days) [37.2% reduction (*p < * 0.05)] infections by *Clostridium difficile* and multidrug-resistant microorganisms were identified.
Marcus et al. (27)	Controlled, non-randomized, cross-over clinical trial. Control group: 54 patients, 4,050 hospitalization days; Intervention group: 58 patients, 4,159 hospitalization days.	Two wards for chronic patients dependent on mechanical ventilation in a long-term hospital (country not specified). Follow-up period: from February to September 2015 (two consecutive 3-month periods, separated by a washout month).	Control group: unimpregnated (not specified) textiles; Intervention group: textiles (not specified) impregnated with copper oxide.	Sheets, bath towels and clothing used by patients during the period of hospitalization.	Control and intervention group: the unimpregnated and impregnated textiles were subjected to the washing process together and in the same way (not specified).	Assessment of HAIs based on the following indicators: days of fever (axillary temperature > 37.6°C); antibiotic treatment initiation events; days of antibiotic treatment; defined daily dose of antibiotics. The method to assess adverse events was not described.	Assessment of the HAI rates: in the control and intervention groups, 188 (46.42 per 1,000 hospitalization days) and 86 (20.68 per 1,000 hospitalization days) days of fever [55.5% reduction (*p < * 0.0001)] were identified, respectively; as well as 95 (23.46 per 1,000 hospitalization days) and 69 (16.59 per 1,000 hospitalization days) antibiotic treatment initiation events [29.3% reduction (*p =* 0.002)]; 689 (170.12 per 1,000 hospitalization days) and 545 (131.04 per 1,000 hospitalization days) days of antibiotic treatment [23% reduction (*p < * 0.0001)]; and 845 (208.6 per 1,000 hospitalization days) and 629 (151.2 per 1,000 hospitalization days) of a defined daily dose of antibiotics [27.5% reduction (*p < * 0.0001)]. Assessment of adverse events: no adverse events were identified.
Tahir et al. (28)	Controlled, non-randomized clinical trial. Sample size: 3 patients.	ICU of a local hospital (country not specified) Follow-up period (not specified) lasting a total of 3 days.	Control group: unimpregnated cotton textiles; Intervention group 1: cotton textiles impregnated with titanium nanoparticles; Intervention group 2: cotton textiles impregnated with silver-doped titanium nanoparticles.	Bed linen (not specified) used by patients during the hospitalization period^†^.	Control group, intervention groups 1 and 2: not specified. The bed linen was subjected to the autoclave sterilization process before being provided to the patients.	Assessment of microbial load: collection of microbiological samples at three sites of each of the bed linen sections.	Microbiological assessment: a mean microbial load higher than 200 CFU/10 cm^2^ was identified at a collection site, and between 150 and 200 CFU/10 cm^2^ in two collection sites in the sections belonging to the control group; a mean microbial load from 50 to 100 CFU/10 cm^2^ was found in the three collection sites in the sections belonging to intervention group 1; and a mean microbial load from 50 to 100 CFU/10 cm^2^ was identified in two collection sites, and between 0 and 50 CFU/10 cm^2^ in a collection site in the sections belonging to intervention group 2.
Argirova et al. (29)	Controlled, non-randomized clinical trial. Control group: 16 patients; Intervention group: 21 patients.	Burns Department of the “Nikolai Ivanovich Pirogov” Multidisciplinary University Hospital for Active Treatment and Emergency Medicine, Bulgaria. Follow-up period: from May to August 2013.	Control group: unimpregnated cotton and polyester textiles; Intervention group: textiles (not specified) impregnated with zinc oxide.	Sheets, pillowcases, blankets and clothing (aprons) used by patients during the hospitalization period.	Control and intervention groups: textiles subjected to the washing process with neutral detergent, at a temperature of 60°C for 60 min. In addition, the textiles were subjected to the sterilization process (not specified) before being provided to the patients.	Assessment of microbial load: collection of microbiological samples in unspecified regions of the control and intervention textiles, before and after 12 hours of use by patients on the first, fourth and seventh evaluation day. The method to assess adverse events was not described^‡^.	Microbiological assessment: after 12 h of use of the textiles by the patients on the first, fourth and seventh evaluation day, respectively, the following microbial loads were identified: from 1 to 3.5 CFU/cm^2^ in 50%, 18.8% and 12.5% of the control group samples, and in 33.3%, 42.9% and 33.3% of the intervention group samples; from 3.5 to 17 CFU/cm^2^ in 43.8%, 43.8% and 62.5% of the control group samples, and in 14.3%, 28.6% and 28.6% of the intervention group samples; from 17 to 58 CFU/cm^2^ in 0%, 25% and 31.3% of the control group samples and in 0%, 4.8% and 9.5% of the intervention group samples (statistical significance and *p*-values not fully presented). Assessment of adverse events: pruritus, erythema and rash were identified in participants from both groups [(total incidence data not specified) (differences not statistically significant (*p*-values not shown)].
Openshaw et al. (30)	Quasi-experimental study. Control period: before and after use, respectively: 454 and 409 samples of sheets, and 466 and 310 samples of aprons. Intervention period: before and after use, respectively: 457 and 394 samples of sheets, and 459 and 303 samples of aprons.	Three community hospitals (country not specified). Control period: from August to September 2015; Intervention period: from December 2015 to January 2016.	Control period: unimpregnated textiles (not specified); Intervention period: textiles (not specified) impregnated with silver ions.	Sheets and clothing (aprons) used by patients during the hospitalization period.	Control period: not specified; Intervention period: textiles subjected to the washing process (not specified), followed by treatment with ionic silver.	Assessment of microbial load: collection of microbiological samples in the lower and upper areas (to the central line) of the sheets, and in the suprapubic and central region of the frontal thorax of the aprons, before and after use by the patients.	Microbiological assessment: before use by the patients: mean reductions of 89% (*p < * 0.0001) and 88% (*p < * 0.0001) CFU of aerobic bacteria in the aprons and sheets, respectively, were identified in the intervention period samples in relation to those corresponding to the control period. After use by the patients: mean reductions of 45% (*p < * 0.0001) and 30% (*p =* 0.0001) CFU of aerobic bacteria in the aprons and sheets, respectively, were identified in the intervention period samples in relation to those corresponding to the control period.
Lazary et al. (31)	Quasi-experimental study. Follow-up period A: 57 patients, 4,337 hospitalization days; Follow-up period B: 51 patients, 3,940 hospitalization days.	Long-term ward for patients with severe head injuries (country not specified). Follow-up period A: from December 2010 to June 2011; Follow-up period B: from December 2011 to June 2012.	Follow-up period A: unimpregnated textiles (not specified); Follow-up period B: textiles (not specified) impregnated with copper oxide.	Sheets, pillowcases, bath towels and clothing (shirts, pants, aprons and robes) used by patients during the hospitalization period.	Follow-up periods A and B: unimpregnated and impregnated textiles were subjected to the washing process in the same way (not specified).	Assessment of microbial load: collection of microbiological samples in the region of the sheets in contact with the upper back of the patients, from 6 to 7 hours after use. Assessment of HAI rates based on theoretical frameworks^c^, ^d^ and on the following indicators: days of fever (body temperature > 38.5°C), antibiotic administration events, and total days of antibiotic administration.	Microbiological assessment: approximately 50% (*p =* 0.005) and 46% (*p =* 0.047) lower Gram-positive and Gram-negative bacterial loads were identified, respectively, in samples from period B in relation to those from period A. Assessment of HAI rates: in follow-up periods A and B, 27.4 and 20.8 HAIs per 1,000 hospitalization days [24% reduction (*p =* 0.046)] were identified; as well as 13.4 and 7.1 days of fever per 1,000 hospitalization days [47% reduction (*p =* 0.0085)]; 21.44 and 16.5 antibiotic administration events per 1,000 hospitalization days [23% reduction (*p =* 0.052)]; and 382.7 and 257.1 total days of antibiotic administration per 1,000 hospitalization days [2.8% reduction (*p < * 0.0001)].
Gabbay et al. (32)	Quasi-experimental study. Sample size in the assessment of microbial load: 30 patients. Sample size in the assessment of adverse events: 100 patients.	Ward of a General Hospital (country not specified). Follow-up period not reported.	Control period: unimpregnated textiles (not specified); Intervention period: cotton textiles impregnated with copper oxide.	Sheets used by patients during the hospitalization period.	Not specified.	Assessment of microbial load: collection of microbiological samples in the regions of the hospital linen that were in contact with the patients' feet after use during the night. The assessment method for adverse events was through a clinical evaluation by specialists.	Microbiological assessment: mean (and standard deviation) values of 21,909 (3,134) CFUs/ml were identified in the control period samples, and of 13,182 (2,863) CFUs/ml in the intervention period samples (*p < * 0.05). Assessment of adverse events: no adverse events were identified.

ICU, Intensive Care Unit; USA, United States of America; HAIs, Healthcare-Associated Infections; CFUs, Colony Forming Units.

* The study has two phases; however, phase two was not integrated into the characterization table or into the methodological quality analysis due to Reason 5;

† Bed linen was divided into three sections, so that each section was part of one of the groups (control, intervention 1 and 2);

‡ The study reports the analysis of the participants' laboratory tests (hematological and biochemical), but the results with parameter deviations were related to the patients' clinical/surgical conditions.

a Centers for Disease Control and Prevention. *Surveillance Definition of Healthcare-Associated Infection and Criteria for Specific Types of Infections in the Acute Care Setting*. CDC (2013).

b Centers for Disease Control and Prevention. *Chapter 2: Identifying Healthcare-Associated Infections (HAIs) for NHSN surveillance*. CDC (2017).

c Embry FC, Chinnes LF. Draft definitions for surveillance of infections in home health care. *Am J Infect Control*. (2000) 28:449–53. 10.1067/mic.2000.112150.

d Stone ND, Ashraf MS, Calder J, Crnich CJ, Crossley K, Drinka PJ, et al. Surveillance definitions of infections in long-term care facilities: revisiting the McGeer criteria. *Infect Control Hosp Epidemiol*. (2012) 33:965–77. 10.1086/667743.

**Chart 2:** Characterization of the studies selected in which the textiles impregnated with antimicrobial substances were used by health professionals during their respective work shifts.

**Identification of the study**	**Characteristics of the study**		**Characteristics of the experiment**			**Assessment method**	**Results**
	**Design and sample size**	**Locus/Country and follow-up period**	**Types of textiles and antimicrobial substances**	**Applicability in health service**	**Hygiene settings of the textiles**		
Salazar-Vargas et al. (33)	Quasi-experimental study (open comparative), cross-over. Sample size: 10 health professionals.	Three wards of the Dr. José Eleuterio González University Hospital, Mexico. Follow-up period: from January to February 2019.	First intervention: sterile textiles (not specified); Second intervention: sterile textiles (not specified), used by the participants after body hygiene with cloths impregnated with 2% chlorhexidine, without rinsing with water; Third intervention: sterile textiles (not specified) impregnated with chlorhexidine.	Surgical uniforms (two pieces) used by nurses during 12-h work shifts.	Intervention groups 1 and 2: the textiles were subjected to the steam sterilization process with dynamic air removal, before being distributed to the participants; Intervention group 3: the textiles were subjected to the steam sterilization process with dynamic air removal and subsequently impregnated before being distributed to the participants.	Assessment of microbial load: collection of microbiological samples from the surgical uniforms [thoracic region (including pocket) and abdominal region], at the beginning and after six and 12 h of the work shifts. The method to assess adverse events was through self-reports by the participants.	Microbiological assessment: at the beginning and after six and 12 h of the work shifts, the following mean microbial loads were identified: 3.58 (from 0 to 26), 13.69 (from 0 to 104) and 20.22 (from 0 to 118) CFUs in the first intervention; 1.26 (from 0 to 22), 3.93 (from 0 to 12) and 5.36 (from 0 to 18) CFUs in the second intervention; and 0.56 (from 0 to 7), 5.16 (from 0 to 39) and 6.7 (from 0 to 39) CFUs in the third intervention. In general, in the first intervention there was a mean of 12.5 CFUs (from 0 to 118), in the second intervention the mean was 3.5 CFUs (from 0 to 22), and in the third intervention it was 3 CFUs (from 0 to 39). The differences were statistically significant between the first and the second intervention (*p =* 0.003), as well as between the first and the third intervention (*p =* 0.007). The differences were not statistically significant between the second and the third intervention (*p =* 0.067). Assessment of adverse events: generalized itching identified by one participant (excluded from the study) in intervention group 2.
Anderson et al. (34)	Controlled, randomized and cross-over clinical trial. Sample size: 40 health professionals.	Medical and Surgical ICU, Duke University Hospital, USA. Follow-up period: from June 2015 to January 2016.	Control group: unimpregnated cotton and polyester textiles; Intervention group 1: textiles (not specified) impregnated with a complex element compound (not specified) and silver alloy; Intervention group 2: textiles (not specified) impregnated with organosilane-based quaternary ammonium and fluoroacrylate copolymer emulsion.	Surgical uniforms used by nurses during 12-h work shifts.	Control group and intervention groups 1 and 2: the researchers proceeded with washing (not specified) the surgical uniforms, five times. Subsequently, the surgical uniforms were packed in plastic packaging and delivered to the study participants.	Assessment of microbial load: collection of microbiological samples only from the upper part [regions of the right sleeve, pocket (located in the left thoracic region) and abdomen] of the surgical uniforms, before the start and end of 12-h work shifts. The method to assess adverse events was through self-reports by the participants.	Microbiological assessment: between the beginning and the end of the work shifts, the following median increases in microbial load were identified: 61.5 CFUs (interquartile range from −3 to 19) in the control group; 73 CFUs (interquartile range from −107 to 194) in intervention group 1; and 54.5 CFUs (interquartile range from −60 to 215) in intervention group 2 (*p =* 0.70). Assessment of adverse events: in the control group and in intervention groups 1 and 2, reports of itching were identified by 2 (5%), 4 (10%) and 12 (30%) participants, respectively (*p =* 0.021); only in intervention groups 1 and 2, reports of erythema or rash were identified by 2 (5%) and 2 (5%) participants, respectively (*p =* 0.54).
Condò et al. (35)	Controlled, non-randomized clinical trial. Control group: 42, 50 and 25 health professionals in the pediatric, surgical and long-term wards, respectively; Intervention group: 46, 43 and 37 health professionals in the pediatric, surgical and long-term wards, respectively;	Pediatric, surgical and long-term wards of the University Hospital of Modena, Italy. Follow-up period not reported.	Control group: unimpregnated textiles (not specified); Intervention group: cotton and polyester textiles impregnated with silver.	Hospital uniforms used by physicians, nurses and health assistants during their respective work shifts.	Not specified.	Assessment of microbial load: collection of microbiological samples from the three uniform pockets, before and after the end of the work shifts.	Microbiological assessment: in the control and intervention groups, the mean CFU ratios (t_0_/t_1_) identified before (t_0_) and after (t_1_) the end of the work shift were as follows: 0.58 and 0.72 CFUs in samples from uniform used in the pediatric ward; 0.49 and 0.46 CFUs in samples from uniform used in the surgical ward; and 0.57 and 0.77 CFUs in samples from uniform used in the long-term ward.
Everson et al. (36)	Controlled, randomized and cross-over clinical trial. Sample size: 17 health professionals.	Inpatient ward for patients with infectious diseases at the Henry Ford Hospital, USA. Follow-up period: from March to May 2012.	Control group: unimpregnated polyester textiles. Intervention group: textiles (not specified) impregnated with silver.	Short coats used by resident physicians for seven consecutive days.	Not specified.	Assessment of microbial load: collection of microbiological samples from the short coats [sleeve region (dominant hand side), edge of the pocket (near the hip) and middle of the back], before delivery to the participants and immediately after removal on the seventh day of use.	Microbiological assessment: before delivery of the short coats, the following mean microbial loads were identified: 1.07 log CFUs/ml in the control group samples, and 0.73 log CFUs/ml (*p =* 0.059) in the intervention group samples. After the seventh day wearing the short coats, the following mean microbial loads were identified: 2.53 log CFUs/ml in the control group samples, and 2.12 log CFUs/ml (*p =* 0.011) in the intervention group samples.
Boutin et al. (37)	Controlled, randomized and cross-over clinical trial. Sample size: 90 health professionals.	IMCU and ICU for adults at the University of Maryland Medical Center, USA. Follow-up period not reported.	Control group: unimpregnated textiles (not specified); Intervention group: textiles (not specified) impregnated with chitosan and dimethylol dimethyl hydantoin.	Hospital uniforms (top and bottom) used by nurses and patient care technicians during 12-h work shifts.	Control and intervention groups: the study participants were instructed to perform the standard (usual) washing of the hospital uniforms at their homes.	Assessment of microbial load: collection of microbiological samples from the upper part (frontal region of the chest to the pelvic girdle, and near the umbilical scar) and lower part (frontal region of both thighs) of the hospital uniforms, in the last 4 h work shifts. The method to assess adverse events was not described.	Microbiological assessment: mean microbial loads of 52 CFUs and 49 CFUs (*p =* 0.67) were identified in the control and intervention groups, respectively. Assessment of adverse events: no adverse events were identified.
Burden et al. (38)	Randomized controlled clinical trial. Control group: 35 health professionals; Intervention group A: 35 health professionals; Intervention Group B: 35 health professionals.	Internal Medicine units from Denver Health, USA. Follow-up period: from March to August 2012.	Control group: unimpregnated cotton and polyester textiles; Intervention group A^*^; Intervention group B: cotton and polyester textiles, impregnated with two patented antimicrobial chemicals (not specified) and silver.	Surgical uniforms (shirt and pants) used by physicians, resident physicians, medical assistants, nurses and clinical nurses during 8-hour work shifts.	Not specified.	Assessment of microbial load: collection of microbiological samples from the shirt [pocket and cuff region of the sleeve (dominant side)] and pants [middle of the thigh region (dominant side)] of the surgical uniforms, after the end of 8-hour work shifts. The method to assess adverse events was through self-reports by the participants.	Microbiological assessment: in general, a median of 99 CFUs (interquartile range from 66 to 182) was identified in the control group samples; and of 138 CFUs (interquartile range from 62 to 274) in the intervention group B samples (*p =* 0.36). Assessment of adverse events: in intervention group B, reports of itching by 1 participant and erythema by 1 participant were identified.
Bearman et al. (39)	Controlled, randomized and cross-over clinical trial. Sample size: 32 health professionals.	ICU of an Academic Medical Center (country not specified) Follow-up period (not specified) lasting a total of 4 months.	Control group: unimpregnated textiles (not specified); Intervention group 2: textiles (unspecified) impregnated with organosilane-based quaternary ammonium and fluoroacrylate copolymer emulsion.	Surgical uniforms (shirt and pants) used by health professionals.	The study protocol provided for the use of four surgical uniforms (two per research group), and each uniform would be used over four consecutive weeks. The participants were instructed to wash the uniforms in hot water with non-bleaching detergent.	Assessment of microbial load: collection of microbiological samples from the pockets (located in the abdominal region) of the shirt and the pocket of the pants of the surgical uniforms, weekly, before the beginning and after the end of the work shift.	Microbiological assessment: in general, in the control and intervention groups, respectively, the mean microbial loads were as follows: Methicillin-resistant *Staphylococcus aureus* presented 11.35 and 7.54 log CFUs (*p =* 0.0056) in the shirt pockets, and 11.84 and 6.71 log CFUs (*p =* 0.0002) in the pants pockets; Vancomycin-resistant *Enterococcus* presented 12.27 and 12.68 log CFUs (*p =* 0.9013) in the shirt pockets, and 12.68 and 0 log CFUs (insufficient representative sample size to calculate p-value) in the pants pockets; gram-negative bacteria (*Escherichia coli, Serratia marcescens* and *Klebsiella p*) presented 10.36 and 9.14 log CFUs (*p =* 0.7569) in the shirt pockets, and 13.02 and 4.41 log CFUs (insufficient representative sample size to calculate *p*-value) in the pants pockets.
Romanò et al. (40)	Controlled, randomized, cross-over and clinical trial. Sample size: 10 health professionals.	The locus and country where the study was developed were not specified. Follow-up period: from March to June 2010.	Control group: unimpregnated cotton and polyester textiles; Intervention group: cotton and polyester textiles, impregnated with quaternary ammonium salts, aromatic and aliphatic alcohols, and isothiazolone derivatives.	Short coats used by physicians for seven consecutive days.	Not specified.	Assessment of microbial load: collection of microbiological samples from the coats [on both sides of the thoracic region, pockets (located in the abdominal region) and sleeves], before and after seven days of use.	Microbiological assessment: in general, in the control and intervention groups, the following mean microbial loads were identified: 213 (from 40 to 360) and 45 (from 5 to 81) CFUs/30 cm^2^ (*p =* 0.03) in samples from the right thoracic region; 296 (from 75 to 400) and 66 (from 10 to 130) CFUs/30 cm^2^ (*p =* 0.02) in samples from the left thoracic region; 452 (from 90 to 780) and 75 (from 25 to 140) CFUs/30 cm^2^ (*p =* 0.01) in samples from the right sleeve region; 1,006 (from 155 to 1,600) and 133 (from 61 to 200) CFUs/30 cm^2^ (*p =* 0.01) in samples from the left sleeve region; 596 (from 115 to 900) and 132 (from 57 to 184) CFUs/30 cm^2^ (*p =* 0.03) in samples from the right pocket region; and 896 (from 390 to 1,275) and 217 (from 100 to 400) CFUs/30 cm^2^ (*p =* 0.01) in samples from the left pocket region.
Groß et al. (41)	Quasi-experimental and cross-over (pilot) study. Sample size: 10 health professionals.	Patient transportation and ambulance company (country not specified). Follow-up period: from January to February 2010.	Follow-up period for the 1st and 3rd weeks: unimpregnated textiles (not specified); Follow-up period for the 2nd and 4th weeks: textiles (not specified) impregnated with silver.	Uniforms of a patient transportation and ambulance company (jacket and pants) used by health professionals.	Follow-up period for the 1st and 3rd weeks, and for the 2nd and 4th weeks: at the beginning of each follow-up week, the uniforms were subjected to the washing process (not specified) in the laundry room, and subsequently packed in plastic packaging.	Assessment of microbial load: collection of microbiological samples from the jackets (right and left frontal region, and lower region of the right sleeve) and pants (right thigh region) of the uniforms, before the first work shift of the follow-up week, and after the end of the third and seventh working days of the follow-up week, with a one-hour interval after removal of the uniforms.	Microbiological assessment: the following was identified before the first work shift and after the end of the third and seventh working days of the follow-up weeks, respectively: 16, 52.7 and 69 CFUs (on average) in unimpregnated jackets, when compared to 20.6 (*p =* 0.542), 199 (*p < * 0.001) and 162.1 (*p < * 0.002) CFUs (on average) in impregnated jackets, in addition to 40.5, 218.5 and 237.1 CFUs (on average) in unimpregnated pants, when compared to 3.3 (*p =* 0.613), 429.5 (*p =* 0.127) and 172.6 (*p =* 0.111) CFUs (on average) in impregnated pants.
Renaud et al. (42)	Controlled, non-randomized and cross-over clinical trial. Sample size: 12 health professionals.	Oncology Surgery Unit of the Léon Bérard Multidisciplinary Center, and ICU of a Military Hospital, France. Follow-up period not reported.	Control group: unimpregnated textiles (not specified); Intervention group: cotton and polyester textiles, impregnated with sodium, silver and copper aluminosilicate.	Hospital uniforms used by nurses and nursing assistants^†^.	Control and intervention groups: the uniforms were subjected to a sterilization process (not specified) before the beginning of the experiment.	Assessment of microbial load: collection of microbiological samples from the hospital uniforms (in the lateral regions belonging to the control and intervention groups), after the end of the work shift (on average 8 h in the Oncology Surgery Unit, and 12 h in the ICU).	Microbiological assessment: in the Oncology Surgery Unit and in the ICU, the following mean values were identified, respectively: 60 (from 4 to 16) and 65 (from 0 to 134) CFUs/25 cm^2^ in the control group samples, and 46 (from 13 to 79) (*p =* 0.057) and 40 (from 6 to 74) (*p =* 0.025) CFUs/25 cm^2^ in the intervention group samples. In general, a 30% lower CFU count was identified in the intervention group samples, when compared to the control group (*p*-value not shown).

CFUs, Colony Forming Units; ICU, Intensive Care Unit; USA, United States of America; IMCU, Intermediate Care Unit.

* The study presents the control group and intervention groups A and B; however, intervention group A was not included in the characterization table or in the analysis of methodological quality, due to Reason 2;

† The hospital uniforms devoid of impregnation had one of their sides (right or left) sewn with a 2 cm^2^ fragment of impregnated textile; therefore, the unmodified side and the modified side belonged to the control and intervention groups, respectively.

**Chart 3:** Characterization of the studies selected where the textiles impregnated with antimicrobial substances were used in inanimate healthcare environments.

**Identification of the study**	**Characteristics of the study**		**Characteristics of the experiment**			**Assessment method**	**Results**
	**Design and sample size**	**Locus/Country and follow-up period**	**Types of textiles and antimicrobial substances**	**Applicability in health service**	**Hygiene settings of the textiles**		
Wilson et al. (43)	Randomized controlled clinical trial. Sample size (not specified in relation to the control group and intervention groups 1 and 2) totaling 45 privacy curtains (including six follow-up losses).	Surgical and Neurological ICU of the Hospital and Clinics of the University of Iowa, USA. Follow-up period: July 2018.	Control group: unimpregnated polyester textiles. Intervention group 1: polyester textiles impregnated with halamine; Intervention group 2: polyester textiles impregnated with halamine (before and after spraying with sodium hypochlorite).	Privacy curtains hanging around the patients' beds in the health services.	Control group and intervention group 1: not specified; Intervention group 2: the curtains were sprayed twice a week with disinfectant spray based on sodium hypochlorite.	Assessment of microbial load: collection of microbiological samples from the front edge (surface not specified) of the privacy curtains, twice a week.	Microbiological assessment: after the end of the follow-up period, the mean microbial loads identified were as follows: from 30 to 40 CFUs in the control group samples; approximately 30 CFUs in the intervention group 1 samples; from 10 to 20 CFUs in the intervention group 2 samples before spraying with sodium hypochlorite; and from 0 to 10 CFUs in the intervention group 2 samples after spraying with sodium hypochlorite. The difference in the mean microbial load between the control group and intervention group 1 samples and between the control group and intervention group 2 samples before spraying was not statistically significant (*p*-values not shown). The difference in the mean CFU microbial load between the control group and intervention group 2 samples after spraying was statistically significant (*p*-value not shown).
Luk et al. (44)	Controlled, non-randomized clinical trial. Control group: 261 privacy curtains; Intervention group A: 46 privacy curtains; Intervention group B: 14 privacy curtains.	Medical, surgical, neurosurgical, orthopedic and rehabilitation units from 10 hospitals, China. Follow-up period: from November 2016 to November 2017.	Control group: unimpregnated polyester textiles. Intervention group A: NWF textiles impregnated with silver additives; Intervention group B: textiles (not specified) impregnated with quaternary ammonium chloride and polyorganosiloxane.	Privacy curtains hanging around the patients' beds in the health services.	Control group: according to the policies (not specified) of each health service; Intervention groups A and B: the privacy curtains were disposable, being replaced every 3–6 months, according to the manufacturers' recommendations (not specified) or after hospital discharge of patients contaminated/infected with multidrug-resistant microorganisms, who occupied the beds where these curtains were allocated.	Assessment of microbial load: collection of microbiological samples from the front edges (of both surfaces) of the privacy curtains, twice a week over the first two follow-up weeks, and once a week over the subsequent follow-up weeks.	Microbiological assessment: in the rooms with patients contaminated/infected with multidrug-resistant microorganisms, the control group samples presented a mean of 27.57 (standard deviation of 74.26) CFUs/100 cm^2^, when compared to the intervention group A samples, which presented a mean of 52.35 (and standard deviation of 117.01) CFUs/100 cm^2^ (*p =* 0.042). In this configuration, the privacy curtains from group B were not allocated. In the ward cubicles, the control group samples presented a mean of 57.23 (standard deviation 102.55) CFUs/100 cm^2^, when compared to the intervention group A samples, which had a mean of 86.98 (standard deviation 153.84) CFUs/100 cm^2^ (*p < * 0.001), and to the intervention group B samples, with a mean of 1.41 (standard deviation 13.28) CFUs/100 cm^2^ (*p < * 0.001).
Kotsanas et al. (45)	Quasi-experimental study. Sample size: 14 privacy curtains.	ICU of the Dandenong Hospital, Australia. Follow-up period: from December 2012 to June 2013.	Intervention: polypropylene textiles, impregnated with antibacterial and antifungal chemicals (not specified) and silver nanometers.	Privacy curtains hanging around the patients' beds in the health services.	The privacy curtains were disposable, being replaced every 6 months.	Assessment of microbial load: collection of microbiological samples from the front edges (from both surfaces) of the privacy curtains, once a month.	Microbiological assessment: a median of 3 CFUs (from 0 to 83) was identified in the samples of antimicrobial privacy curtains.

ICU, Intensive Care Unit; USA, United States of America; CFU, Colony Forming Unit; NWF, Non-Woven Fabric.

Charts 4, 5 present the methodological quality assessment (risk of bias) corresponding to these studies, according to the specific tools for each type of design, made available by the JBI.

**Chart 4:** Methodological quality assessment (risk of bias) corresponding to the quasi-experimental studies and non-randomized clinical trials, according to the Checklist for Quasi-Experimental Studies (Non-Randomized Experimental Studies) tool made available by the JBI.

**Studies conducted with/in:**	**Identification**	**Q1**	**Q2**	**Q3**	**Q4**	**Q5**	**Q6**	**Q7**	**Q8**	**Q9**	**“Yes” Score**	**Methodological quality**	**Risk of bias**
Patients	Balachandran et al. (24)	+	?	+	+	+	NA	+	?	+	75%	High	Low
	Madden et al. (25)	+	?	-	-	+	NA	-	?	+	37.5%	Low	High
	Butler (26)	+	?	+	-	+	NA	+	?	+	62.5%	Moderate	Moderate
	Marcus et al. (27)	+	+	+	+	+	NA	+	?	+	87.5%	High	Low
	Tahir et al. (28)	+	+	?	+	-	+	+	?	?	55.6%	Moderate	Moderate
	Argirova et al. (29)	+	?	?	+	+	+	+	?	+	66.7%	Moderate	Moderate
	Openshaw et al. (30)	+	?	?	-	+	NA	+	?	+	50%	Moderate	Moderate
	Lazary et al. (31)	+	+	+	-	+	NA	+	?	+	75%	High	Low
	Gabbay et al. (32)	+	+	?	-	-	+	+	?	+	55.6%	Moderate	Moderate
Healthcare professionals	Salazar-Vargas et al. (33)	+	+	?	-	+	+	+	?	+	66.7%	Moderate	Moderate
	Condò et al. (35)	+	?	?	+	+	?	+	?	?	44.4%	Low	High
	Groß et al. (41)	+	+	?	-	+	+	+	?	+	66.7%	Moderate	Moderate
	Renaud et al. (42)	+	+	?	+	-	?	+	?	+	55.6%	Moderate	Moderate
Inanimate environments of healthcare	Luk et al. (44)	+	?	?	+	+	-	+	?	+	55.6%	Moderate	Moderate
	Kotsanas et al. (45)	+	NA	NA	-	+	+	NA	?	NA	60%	Moderate	Moderate

Q1: Is it clear in the study what is the “cause” and what is the “effect” (i.e., there is no confusion about which variable comes first)?; Q2: Were the participants included in any comparisons similar?; Q3: Were the participants included in any comparisons receiving similar treatment/care, other than the exposure or intervention of interest?; Q4: Was there a control group?; Q5: Were there multiple measurements of the outcome both pre and post the intervention/exposure?; Q6: Was follow up complete and if not, were differences between groups in terms of their follow up adequately described and analyzed?; Q7: Were the outcomes of participants included in any comparisons measured in the same way?; Q8: Were outcomes measured in a reliable way?; Q9: Was appropriate statistical analysis used?; (+): Yes; (−): No; (?): Unclear; (NA): Not Applicable.

**Chart 5:** Methodological quality assessment (risk of bias) corresponding to the randomized clinical trials, according to the checklist for randomized controlled trials tool made available by the JBI.

**Studies conducted with/in:**	**Identification**	**Q1**	**Q2**	**Q3**	**Q4**	**Q5**	**Q6**	**Q7**	**Q8**	**Q9**	**Q10**	**Q11**	**Q12**	**Q13**	**“Yes” Score**	**Methodological quality**	**Risk of bias**
Patients	Marik et al. (23)	?	+	+	-	+	+	+	NA	+	+	?	+	+	75%	High	Low
Healthcare professionals	Anderson et al. (34)	?	?	+	+	-	+	+	+	+	+	?	+	+	69,2%	Moderate	Moderate
	Everson et al. (36)	?	?	+	-	-	-	?	+	+	+	?	+	+	46.2%	Low	High
	Boutin et al. (37)	+	?	+	+	-	-	?	+	+	+	?	?	+	53.8%	Moderate	Moderate
	Burden et al. (38)	+	?	?	-	-	-	+	+	+	+	?	+	+	53.8%	Moderate	Moderate
	Bearman et al. (39)	?	?	+	+	-	+	+	-	-	+	?	+	+	53.8%	Moderate	Moderate
	Romanò et al. (40)	?	?	+	+	-	+	?	+	+	+	?	+	+	61.5%	Moderate	Moderate
Inanimate environments of healthcare	Wilson et al. (43)	+	?	?	+	-	-	?	-	+	+	?	+	+	46.2%	Low	High

Q1: Was true randomization used for assignment of participants to treatment groups?; Q2: Was allocation to treatment groups concealed?; Q3: Were treatment groups similar at the baseline?; Q4: Were participants blind to treatment assignment?; Q5: Were those delivering treatment blind to treatment assignment?; Q6: Were outcomes assessors blind to treatment assignment?; Q7: Were treatment groups treated identically other than the intervention of interest?; Q8: Was follow up complete and if not, were differences between groups in terms of their follow up adequately described and analyzed?; Q9: Were participants analyzed in the groups to which they were randomized?; Q10: Were outcomes measured in the same way for treatment groups?; Q11: Were outcomes measured in a reliable way?; Q12: Was appropriate statistical analysis used?; Q13: Was the trial design appropriate, and any deviations from the standard RCT design (individual randomization, parallel groups) accounted for in the conduct and analysis of the trial?; (+): Yes; (−): No; (?): Unclear; (NA): Not Applicable.

Among the 10 studies in which the textiles impregnated with antimicrobial substances were used by patients during the hospitalization period, five and four studies, respectively, only evaluated the HAI rates and the microbial load in these textiles, while only one study evaluated both the HAI rates and the microbial load in these textiles (Chart 1).

In five of the six studies in which copper was the impregnating substance of the textiles used by the patients, there was efficacy in reducing the microbial load of these textiles and/or the HAI rates, when compared to conventional textiles (even if this difference was not always considered statistically significant), and their methodological quality was considered moderate (moderate risk of bias) (26, 32) or high (low risk of bias) (23, 27, 31) while the only study conducted under these same configurations that did not result in efficacy in reducing the HAI rates presented low methodological quality (high risk of bias) (25).

In the two studies where silver was the impregnating substance of the textiles used by the patients, there was efficacy in reducing the microbial load of these textiles and the HAI rates, when compared to conventional textiles, and their methodological quality was considered moderate (moderate risk of bias) (30) and high (low risk of bias) (24).

As for the other two studies in which zinc oxide, (29) and titanium nanoparticles and silver-doped titanium nanoparticles (together), (28) were the impregnating substances of the textiles used by the patients, there was efficacy in reducing the microbial load of these textiles, when compared to conventional textiles (even if the statistical significance was not always evaluated or presented), and their methodological quality was considered moderate (moderate risk of bias).

Among these 10 studies in which the textiles impregnated with antimicrobial substances were used by the patients during the hospitalization period, only three evaluated the occurrence of adverse events presented by the participants.

No adverse events were identified in two studies where copper was the impregnating substance of the textiles used by the patients (27, 32). In the study where zinc oxide was the impregnating substance of the textiles used by the patients, itching, erythema and rash were identified in the participants belonging to the control and intervention groups (29).

All 10 studies in which the textiles impregnated with antimicrobial substances were used by health professionals during their respective work shifts only evaluated the microbial load in these textiles (**Chart 2**).

In three of the five studies where silver was the impregnating substance for the textiles used by health professionals, there was no efficacy in reducing the microbial load of these textiles, when compared to conventional textiles, and their methodological quality was considered moderate (moderate risk of bias) (34, 38, 41). while the other two studies conducted under these same configurations that resulted in efficacy in reducing the microbial load of these textiles when compared to conventional textiles (even if this difference was not always considered statistically significant, or even if the statistical significance was not always evaluated or presented), had low methodological quality (high risk of bias) (35, 36).

In the two studies where quaternary ammonium was the impregnating substance for the textiles used by health professionals, there was efficacy in reducing the microbial load of these textiles, when compared to conventional textiles (even if this difference was not always considered statistically significant), and their methodological quality was considered moderate (moderate risk of bias) (34, 39).

In the study where chlorhexidine was the impregnating substance of the textiles used by health professionals, there was efficacy in reducing the microbial load of these textiles, when compared to conventional textiles, and its methodological quality was considered moderate (moderate risk of bias) (33).

In the three studies where silver and copper (together), (42) quaternary ammonium, alcohols and isothiazolone derivatives (together), (40) and chitosan and dimethylol dimethyl hydantoin (together), (37) were the impregnation substances of the textiles used by health professionals, there was efficacy in reducing the microbial load of these textiles, when compared to conventional textiles (even if this difference was not always was considered statistically significant), and their methodological quality was considered moderate (moderate risk of bias).

Among these 10 studies in which the textiles impregnated with antimicrobial substances were used by health professionals during their respective work shifts, only four evaluated the occurrence of adverse events presented by the participants.

In the studies where chlorhexidine, (33) and chitosan and dimethylol dimethyl hydantoin (together), (37) were the impregnating substances in the textiles used by health professionals, no adverse events were identified (in the participants of the interventions of interest). In two studies where silver was the substance employed to impregnate the textiles used by health professionals, itching and erythema were identified in participants from the intervention group (38), in addition to itching in participants belonging to the control and intervention groups and erythema or rash in those from the intervention group (34). In a study where quaternary ammonium was the substance employed to impregnate the textiles used by health professionals, itching was identified in participants belonging to the control and intervention groups, and erythema or skin rash in those from the intervention group (34).

All three studies where the textiles impregnated with antimicrobial substances were used in inanimate healthcare environments only assessed the microbial load in these textiles (Chart 3).

In one of the two studies where silver was the substance employed to impregnate the textiles used in inanimate healthcare environments, there was no control group or period that allowed comparing results, (45) whereas, in the other study there was no efficacy in reducing the microbial load of these textiles, when compared to conventional textiles (44), with the methodological quality of both considered as moderate (moderate risk of bias).

In the study where halamine was the substance employed to impregnate the textiles used in inanimate healthcare environments, there was efficacy in reducing the microbial load of these textiles, when compared to conventional textiles (even though this difference was not always considered statistically significant) and its methodological quality was considered low (high risk of bias) (43).

In the study where quaternary ammonium was the substance employed to impregnate the textiles used in inanimate healthcare environments, there was efficacy in reducing the microbial load of these textiles, when compared to conventional textiles, and its methodological quality was considered moderate (moderate risk of bias) (44).

In these three studies where the textiles impregnated with antimicrobial substances were used in inanimate healthcare environments, no assessments of the occurrence of adverse events in individuals (patients and health professionals) who came into contact with these textiles were performed.

## Discussion

Development of this systematic review allowed evidencing that the composition of the scientific literature on textiles impregnated with antimicrobial substances is massively based on laboratory experiments, especially *in vitro*, and that there are still few studies conducted in real healthcare conditions, among which only a small percentage was able to recruit significant sample sizes for their respective control and intervention groups/periods, took place in multiple centers, or conducted the control and intervention experimental analyses concomitantly or in parallel follow-up periods, allowing extrapolation of the results to other similar contexts.

With regard to the methodological configurations of the studies selected to comprise this review, it is evidenced how many possibilities there are for combinations between the independent and dependent variables. The independent variables mainly refer to the types of textiles, antimicrobial substances and applicability of the impregnated/antimicrobial textiles in health services. As for the dependent variables, they refer to possible analyses related to microbial contamination in the textiles and to the HAI rates.

In relation to the independent variables, due to the multiple applicability possibilities of the textiles impregnated with antimicrobial substances in health services, it was agreed to subdivide the studies included in this review according to the use configurations of these textiles, so that this use could be by the patients during the hospitalization period, by the health professionals during their respective work shifts, and in inanimate healthcare environments. From the aforementioned, an important gap is highlighted in the scientific literature on the theme, which refers to the absence of studies that address concomitant use of impregnated/antimicrobial textiles by patients, by professionals and in inanimate healthcare environments.

In the studies selected where the hospitalized patients used textiles impregnated with substances with antimicrobial activity (Chart 1), exclusive impregnation with heavy metals is noticed, specifically copper, silver, zinc oxide and titanium nanoparticles in an isolated manner, and silver-doped titanium nanoparticles together. In general, considering methodological quality, after use by the patients, the textiles impregnated with these substances presented lower microbial loads and/or resulted in lower HAI rates when compared to the use of conventional textiles.

In the studies selected where the health professionals used textiles impregnated with substances with antimicrobial activity (**Chart 2**), impregnation took place with silver, quaternary ammonium and chlorhexidine in isolation; silver and copper together; quaternary ammonium, alcohols and isothiazolone derivatives together; and chitosan and dimethylol dimethyl hydantoin together. In general, considering methodological quality, after being used by health professionals, the textiles impregnated with these substances, with the exception of those impregnated with silver in isolation, presented lower microbial loads when compared to the use of conventional textiles.

In the studies selected where inanimate healthcare environments were treated with textiles impregnated with substances with antimicrobial activity (Chart 3), impregnation was with silver, halamine and quaternary ammonium, in isolation. In general, considering methodological quality, after use in inanimate healthcare environments, the textiles impregnated with these substances, with the exception of those impregnated with silver alone, presented lower microbial loads when compared to the use of conventional textiles. It is also worth mentioning that, according to the critical evaluation, the study conducted under these same use and applicability configurations in which the textiles were impregnated with halamine, resulting in a lower microbial load when compared to conventional textiles, obtained low methodological quality, which renders such outcomes questionable.

Heavy metals have antimicrobial properties through multiple mechanisms of action in microbial cells, namely: interference in cell wall synthesis; depolarization of the electrical potential of the plasma membrane; plasma membrane lysis; protein denaturation; and induction of oxidative stress, which can also act in synergy, potentiating antimicrobial activity (46). However, considering the methodological quality of the studies selected in which silver in isolation was impregnated in the textiles used by health professionals or allocated to inanimate healthcare environments, there is no efficacy in reducing the microbial load of these textiles when compared to unimpregnated textiles. Among the hypotheses that can explain this specific ineffectiveness, the probable overestimation of the antimicrobial effect of these textiles by health professionals stands out, mainly in studies with no blinding configurations, causing non-compliance with basic biosafety measures, for example: compromising hand hygiene, leading to more errors during the gowning and degowning processes, and even resulting in failures during decontamination of fomites and their inanimate environments, which corroborates to potentiating microbial contamination, even in the impregnated/antimicrobial textiles themselves.

In addition to the mechanisms of action of the antimicrobial substances impregnated in the textiles, application of different antimicrobials in the impregnation process of the same textile material is noted as a potential perspective, aiming, through the attribution of various antimicrobial properties, to achieve maximum biostatic or biocidal efficacy and, thus, minimize any risk for the development and spread of microbial resistance. However, it can be observed that most of the clinical studies involving the use of impregnated/antimicrobial textiles are still characterized by the absence of detailed information related to the textile materials, the antimicrobial substances and the impregnation method, which hinders replication of these research studies, methodological validation in similar contexts and rapid progress in filling the gaps in this scientific field.

In this context, there is also significant lack of information regarding the hygiene settings of the antimicrobial textiles when used in the clinical practice concerning the products and methods used, as well as the time interval between sanitation procedures. Thus, there is no way to accurately identify the ideal conditions for sanitizing such antimicrobial textiles when used in real healthcare circumstances, aiming to provide maximum durability of the antimicrobial effect and, mainly, at the reduction of dirt, organic matter and microbial load in these textiles.

In relation to the dependent variables of interest in this systematic review, it is observed that microbiological collection on the textile surfaces can be performed through different methods, as well as the analyses related to microbial contamination, in short: specific and unspecific microbial load regarding the type(s) of microorganism(s); microbial load on certain areas of the textile surface; and microbial load before and after use in healthcare services. With regard to the HAI rates, there is also the possibility of identifying them through different methods, either by means of pre-established clinical indicators or according to a theoretical framework adopted, which are subjected to periodic updates; in addition to that, the respective analyses can refer to specific and non-specific HAIs regarding the etiological agent and the tissues, organs or physiological systems affected.

Despite recognizing that the development of each and every infection is a multifactorial process due to the complexity of the epidemiological chain, there is no way to disregard microbial load as a crucial factor in this context (47, 48). For this reason, studies that involve the use of antimicrobial textiles in real healthcare conditions and assess the impacts on the HAI rates, as well as the microbial load in this textiles, are extremely valuable; however, as evidenced in the current review, there is a minimal percentage of studies that have concomitantly conducted such analyses.

Among the factors of concern, the safety of using textiles impregnated with antimicrobial substances must be considered, as adverse events mainly represented by dermatological signs and symptoms can occur in patients and/or health professionals who are in continuous contact with these textiles. However, it is noted that there was no significant investigation of the adverse events presented by the participants of the studies that comprise the current review; therefore, any and all risks to human health cannot be ruled out and deserve greater attention. It is worth noting that, regardless of the antimicrobial substance impregnated in the textile material, its concentration should be seen as a fundamental part in striking a balance between protective functionality, whether biostatic or biocidal, and safety, in relation to non-induction of toxicity and occurrence of adverse events in the users (16).

In addition to that, in clinical studies with no blinding settings for the participants, the nocebo effect should not be ignored, as it is possibly responsible for eventual bumps in the notification rates of self-reported adverse events. Thus, it becomes strictly necessary that, in future studies, this type of evaluation is carried out by specialized and trained professionals whenever possible, through anamnesis together with a physical examination of the patients and health professionals who make use of the impregnated/antimicrobial textiles.

The environmental impacts also deserve due attention since, in the long-, medium- or even short-term, repeated decontamination procedures by washing impregnated textiles can lead to leaching of certain antimicrobials and to consequent contamination of water ecosystems (49). Thus, instead of using synthetic compounds in the textile impregnation field, natural bioactive substances with antimicrobial properties, mainly obtained from medicinal plants and other organic substrates, can serve as a safe and renewable alternative to the environment for biodegradability and sustainability reasons (50, 51). Other strategies can also be listed, such as passive coatings devoid of any antimicrobial properties, on the textile surfaces, which, despite not presenting biocidal or biostatic activity, are responsible for hindering microbial adhesion and are characterized as easily decontaminated (52).

From an economic point of view, there is probable profitability of antimicrobial textiles, mainly when comparing the costs related to their acquisition to those required for the acquisition and application of other substances with antimicrobial activity, commonly used in health services, aiming to promote biosafety. The same profitability can be observed when considering the potential of antimicrobial textiles in preventing and controlling the HAI rates and, consequently, the substantial costs for the treatment of these infections, in addition to the incalculable harms related to morbidity and mortality. Undoubtedly, it is emphasized that economic modeling should be considered as an integral component of the evaluation of future clinical studies in this knowledge area, in order to estimate the true cost-benefit ratio of antimicrobial textiles (53).

Finally, although textiles impregnated with antimicrobials show promising results in terms of reducing microbial load and HAI rates, it is noted that this potential antimicrobial effect should not be overestimated under any circumstance, being indispensable to regulate periodic and standardized decontamination of these resources. Accordingly, innovative and practical methods that allow identifying microbial contamination in textiles (54) can be considered complementary tools for assessing the antimicrobial efficacy of impregnated textiles, also assisting in monitoring microbial resistance, while they can also act as markers of contamination by viable microorganisms on the surfaces of these textiles, even after extended periods of continuous exposure. In this way, effective recovery of microorganisms resistant to antimicrobials impregnated in textile materials is made possible and, consequently, surveillance of cross-resistance, co-resistance and resistance by co-regulation to other antimicrobials, especially pharmacological ones, a fundamental requirement for the safe implementation of impregnated/antimicrobial textiles in healthcare services.

As for the limitations of the current study, it is noted that, given the extensive number of existing databases and Gray Literature materials, as well as the infinite possibilities of search strategies to be developed, considering the countless controlled terms and their respective synonyms, it is impossible to state that a full scan was carried out in the scientific literature on the theme of interest and, consequently, other potentially eligible studies may not have been identified.

In addition to that, the marked heterogeneity of methodological configurations across the studies selected, as well as their methodological quality levels, made it impossible to conduct quantitative syntheses of the results evidenced, which precludes statistical inferences. Finally, as agreed in the review protocol, with the impossibility of performing a quantitative synthesis of the results, certainty of the evidence was not evaluated by means of the GRADE system, thus making it impossible to provide a basis for the elaboration of recommendations and guidelines to be implemented in the clinical practice.

In relation to the contributions of this study, the systematic review design allowed identifying which substances with antimicrobial properties impregnated (either in isolation or together) in textiles, as well as which configurations of their use (by patients, by health professionals or in inanimate environments) in healthcare services, confer efficacy in reducing the microbial load present in these textiles and/or the HAI rates when compared to conventional textiles.

In addition, the comprehensive search in the scientific literature made it possible to diagnose the current scenario on the theme of interest and, thus, to evidence the main gaps that still need to be bridged in order to provide safe and effective use of antimicrobial textiles in healthcare services.

## Conclusions

In the current systematic review, the qualitative synthesis, taking into account the methodological quality of the studies selected, allowed identifying which antimicrobial substances impregnated in textiles used in healthcare services confer efficacy in reducing the microbial load present in these textiles and/or the HAI rates, when compared to conventional textiles.

Among the antimicrobial substances impregnated in textiles and used by patients during the hospitalization period, it can be concluded that copper; silver; zinc oxide; titanium nanoparticles; and silver-doped titanium nanoparticles together; confer efficacy in reducing the microbial load present in these textiles and/or the HAI rates, when compared to conventional textiles.

Among the antimicrobial substances impregnated in textiles used by health professionals during their respective work shifts, it can be concluded that quaternary ammonium; chlorhexidine; silver and copper together; quaternary ammonium, alcohols and isothiazolone derivatives together; and chitosan and dimethylol dimethyl hydantoin together; confer efficacy in reducing the microbial load present in these textiles, when compared to conventional textiles.

Among the antimicrobial substances impregnated in textiles used in inanimate healthcare environments, it can be concluded that quaternary ammonium confers efficacy in reducing the microbial load present in these textiles when compared to conventional textiles.

Due to the scarcity of research studies regarding adverse events presented by the patients and health professionals after using or entering into contact with textiles impregnated with antimicrobial substances, there were difficulties meeting the objective of listing them in the current review. However, in the studies that conducted such analyses, it can be verified that the individuals who used these textiles were not exempt from presenting signs and symptoms of cutaneous toxicity.

When used by patients, copper-impregnated textiles did not induce adverse events, whereas textiles impregnated with zinc oxide induced itching, erythema and/or rash. When used by health professionals, textiles impregnated with chlorhexidine and with chitosan and dimethylol dimethyl hydantoin together did not induce adverse events, whereas textiles impregnated with silver and quaternary ammonium induced pruritus, erythema and/or rash.

## Author contributions

Conceptualization, study design, data acquisition, data analysis, interpretation of results, and drafted the manuscript: GS, LV, HC, AS, EW, DA, and RS. Revised manuscript: GS, LV, AS, and DA. All authors approved the final version and agree to be accountable for all aspects of this work.
