# Supplementary material for: Textiles impregnated with antimicrobial substances in healthcare services: systematic review
Source: Front Public Health. 2023 May 11;11:1130829. doi: 10.3389/fpubh.2023.1130829 (PMC10213888; doi:10.3389/fpubh.2023.1130829)
Supplement: Supplementary file 1 [file Data_Sheet_1.pdf]

Supplementary materials (SM)

SM1: Chart 1 - Search strategies, applied by information sources on October 23rd, 2021, during the process to identify studies in the scientific literature.

Sources of information	Search strategy	Search field	Identified results
MEDLINE	(“Soft Surface” OR “Soft Surfaces” OR Woven OR Wovens OR Nonwoven OR Nonwovens OR Textiles[Mesh] OR Textile OR Textiles OR Fabric OR Fabrics OR “Cotton Fiber”[Mesh] OR “Cotton Fiber” OR “Cotton Fibers” OR “Cotton Fabric” OR “Cotton Fabrics” OR Cotton OR Cottons OR Polyesters[Mesh] OR Polyester OR Polyesters OR “Bedding and Linens”[Mesh] OR Bedding OR Beddings OR Linen OR Linens OR Clothing[Mesh] OR Clothing OR Clothes OR Uniform OR Uniforms OR “Protective Clothing”[Mesh] OR “Protective Clothing” OR “Protective Clothes” OR Scrub OR Scrubs OR Gown OR Gowns OR “White Coat” OR “White Coats” OR “Lab Coat” OR “Lab Coats” OR Curtain OR Curtains) AND (Impregnated OR Impregnation OR Impregnating OR Incorporated OR Incorporation OR Incorporating OR Coated OR Coating OR Finish OR Finished OR Finishing OR Doped) AND (“Self-Disinfecting” OR Antimicrobial OR Antimicrobials OR Microbicide OR Microbicides OR Antibacterial OR Antibacterials OR Bactericide OR Bactericides OR Sporicide OR Sporicides OR Sporicidal OR Sporicidals OR Antifungical OR Antifungicals OR Antifungal OR Antifungals OR Fungicide OR Fungicides OR Antiviral OR Antivirals OR Viricide OR Viricides OR Biocide OR Biocides OR Inactivated OR Inactivating) AND (“Cross Infection”[Mesh] OR “Cross Infection” OR “Cross Infections” OR “Healthcare Associated Infection” OR “Healthcare Associated Infections” OR “Healthcare Acquired Infection” OR “Healthcare Acquired Infections” OR “Nosocomial Infection” OR “Nosocomial Infections” OR “Hospital Acquired Infection” OR “Hospital Acquired Infections” OR “Hospital Infection” OR “Hospital Infections” OR “HAI” OR “HAIs” OR Contamination OR Contaminations OR “Microbial Contamination” OR “Microbial Contaminations” OR “Microbial Load” OR “Microbial Loads” OR “Bacterial Contamination” OR “Bacterial Contaminations” OR “Bacterial Load”[Mesh] OR “Bacterial Load” OR “Bacterial Loads” OR “Bacterial Count” OR “Bacterial Counts” OR Bioburden)	All fields	438
EMBASE	('Soft Surface' OR 'Soft Surfaces' OR Woven OR Wovens OR Nonwoven OR Nonwovens OR 'Textile'/exp OR Textile OR 'Textiles'/exp OR Textiles OR 'Fabric'/exp OR Fabric OR Fabrics OR 'Cotton Fiber'/exp OR 'Cotton Fiber' OR 'Cotton Fibers' OR 'Cotton Fabric'/exp OR 'Cotton Fabric' OR 'Cotton Fabrics' OR 'Cotton'/exp OR Cotton OR Cottons OR 'Polyester'/exp OR Polyester OR 'Polyesters'/exp OR Polyesters OR Bedding OR Beddings OR 'Linen'/exp OR Linen OR Linens OR 'Clothing'/exp OR Clothing OR 'Clothes'/exp OR Clothes OR Uniform OR Uniforms OR 'Protective	All fields	329

	Clothing'/exp OR 'Protective Clothing' OR 'Protective Clothes' OR 'Scrub'/exp OR Scrub OR Scrubs OR 'Gown'/exp OR Gown OR Gowns OR 'White Coat'/exp OR 'White Coat' OR 'White Coats' OR 'Lab Coat' OR 'Lab Coats' OR Curtain OR Curtains) AND (Impregnated OR 'Impregnation'/exp OR Impregnation OR Impregnating OR Incorporated OR 'Incorporation'/exp OR Incorporation OR Incorporating OR Coated OR 'Coating'/exp OR Coating OR Finish OR Finished OR Finishing OR Doped) AND ('Self-Disinfecting' OR 'Antimicrobial'/exp OR Antimicrobial OR 'Antimicrobials'/exp OR Antimicrobials OR 'Microbicide'/exp OR Microbicide OR Microbicides OR 'Antibacterial'/exp OR Antibacterial OR Antibacterials OR 'Bactericide'/exp OR Bactericide OR Bactericides OR Sporicide OR Sporicides OR Sporicidal OR Sporicidals OR Antifungical OR Antifungicals OR 'Antifungal'/exp OR Antifungal OR 'Antifungals'/exp OR Antifungals OR 'Fungicide'/exp OR Fungicide OR 'Fungicides'/exp OR Fungicides OR 'Antiviral'/exp OR Antiviral OR 'Antivirals'/exp OR Antivirals OR Viricide OR Viricides OR 'Biocide'/exp OR Biocide OR Biocides OR Inactivated OR Inactivating) AND ('Cross Infection'/exp OR 'Cross Infection' OR 'Cross Infections' OR 'Healthcare Associated Infection'/exp OR 'Healthcare Associated Infection' OR 'Healthcare Associated Infections' OR 'Healthcare Acquired Infection'/exp OR 'Healthcare Acquired Infection' OR 'Healthcare Acquired Infections' OR 'Nosocomial Infection'/exp OR 'Nosocomial Infection' OR 'Nosocomial Infections' OR 'Hospital Acquired Infection'/exp OR 'Hospital Acquired Infection' OR 'Hospital Acquired Infections' OR 'Hospital Infection'/exp OR 'Hospital Infection' OR 'Hospital Infections' OR 'HAI' OR 'HAIs' OR 'Contamination'/exp OR Contamination OR Contaminations OR 'Microbial Contamination'/exp OR 'Microbial Contamination' OR 'Microbial Contaminations' OR 'Microbial Load'/exp OR 'Microbial Load' OR 'Microbial Loads' OR 'Bacterial Contamination'/exp OR 'Bacterial Contamination' OR 'Bacterial Contaminations' OR 'Bacterial Load'/exp OR 'Bacterial Load' OR 'Bacterial Loads' OR 'Bacterial Count'/exp OR 'Bacterial Count' OR 'Bacterial Counts' OR 'Bioburden'/exp OR Bioburden)		
CINAHL	(“Soft Surface” OR “Soft Surfaces” OR Woven OR Wovens OR Nonwoven OR Nonwovens OR Textile OR Textiles OR Fabric OR Fabrics OR “Cotton Fiber” OR “Cotton Fibers” OR “Cotton Fabric” OR “Cotton Fabrics” OR Cotton OR Cottons OR Polyester OR Polyesters OR Bedding OR Beddings OR Linen OR Linens OR Clothing OR Clothes OR Uniform OR Uniforms OR “Protective Clothing” OR “Protective Clothes” OR Scrub OR Scrubs OR Gown OR Gowns OR “White Coat” OR “White Coats” OR “Lab Coat” OR “Lab Coats” OR Curtain OR Curtains) AND ((Impregnated OR Impregnation OR Impregnating OR Incorporated OR Incorporation OR Incorporating OR Coated OR Coating OR Finish OR Finished OR Finishing OR Doped) OR (“Self-Disinfecting” OR Antimicrobial OR Antimicrobials OR Microbicide OR Microbicides OR Antibacterial OR Antibacterials OR Bactericide OR Bactericides OR Sporicide OR Sporicides OR Sporicidal OR Sporicidals OR Antifungical OR Antifungicals OR Antifungal OR Antifungals OR Fungicide OR Fungicides OR Antiviral OR Antivirals OR Viricide OR Viricides OR Biocide OR Biocides OR Inactivated OR Inactivating)) AND (“Cross Infection” OR “Cross Infections” OR “Healthcare Associated Infection” OR “Healthcare Associated Infections” OR “Healthcare Acquired Infection” OR “Healthcare Acquired Infections” OR “Nosocomial Infection” OR “Nosocomial Infections” OR “Hospital Acquired Infection” OR “Hospital Acquired Infections” OR “Hospital Infection” OR “Hospital Infections” OR “HAI” OR “HAIs” OR Contamination OR Contaminations OR “Microbial Contamination” OR “Microbial	Basic search	189

	Contaminations” OR “Microbial Load” OR “Microbial Loads” OR “Bacterial Contamination” OR “Bacterial Contaminations” OR “Bacterial Load” OR “Bacterial Loads” OR “Bacterial Count” OR “Bacterial Counts” OR Bioburden)		
Web of Science	(“Soft Surface” OR “Soft Surfaces” OR Woven OR Wovens OR Nonwoven OR Nonwovens OR Textile OR Textiles OR Fabric OR Fabrics OR “Cotton Fiber” OR “Cotton Fibers” OR “Cotton Fabric” OR “Cotton Fabrics” OR Cotton OR Cottons OR Polyester OR Polyesters OR Bedding OR Beddings OR Linen OR Linens OR Clothing OR Clothes OR Uniform OR Uniforms OR “Protective Clothing” OR “Protective Clothes” OR Scrub OR Scrubs OR Gown OR Gowns OR “White Coat” OR “White Coats” OR “Lab Coat” OR “Lab Coats” OR Curtain OR Curtains) AND (Impregnated OR Impregnation OR Impregnating OR Incorporated OR Incorporation OR Incorporating OR Coated OR Coating OR Finish OR Finished OR Finishing OR Doped) AND (“Self-Disinfecting” OR Antimicrobial OR Antimicrobials OR Microbicide OR Microbicides OR Antibacterial OR Antibacterials OR Bactericide OR Bactericides OR Sporicide OR Sporicides OR Sporicidal OR Sporicidals OR Antifungical OR Antifungicals OR Antifungal OR Antifungals OR Fungicide OR Fungicides OR Antiviral OR Antivirals OR Viricide OR Viricides OR Biocide OR Biocides OR Inactivated OR Inactivating) AND (“Cross Infection” OR “Cross Infections” OR “Healthcare Associated Infection” OR “Healthcare Associated Infections” OR “Healthcare Acquired Infection” OR “Healthcare Acquired Infections” OR “Nosocomial Infection” OR “Nosocomial Infections” OR “Hospital Acquired Infection” OR “Hospital Acquired Infections” OR “Hospital Infection” OR “Hospital Infections” OR “HAI” OR “HAIs” OR Contamination OR Contaminations OR “Microbial Contamination” OR “Microbial Contaminations” OR “Microbial Load” OR “Microbial Loads” OR “Bacterial Contamination” OR “Bacterial Contaminations” OR “Bacterial Load” OR “Bacterial Loads” OR “Bacterial Count” OR “Bacterial Counts” OR Bioburden)	Topic (searches title, abstract, author keywords, and Keywords Plus)	259
Scopus	(“Soft Surface” OR “Soft Surfaces” OR Woven OR Wovens OR Nonwoven OR Nonwovens OR Textile OR Textiles OR Fabric OR Fabrics OR “Cotton Fiber” OR “Cotton Fibers” OR “Cotton Fabric” OR “Cotton Fabrics” OR Cotton OR Cottons OR Polyester OR Polyesters OR Bedding OR Beddings OR Linen OR Linens OR Clothing OR Clothes OR Uniform OR Uniforms OR “Protective Clothing” OR “Protective Clothes” OR Scrub OR Scrubs OR Gown OR Gowns OR “White Coat” OR “White Coats” OR “Lab Coat” OR “Lab Coats” OR Curtain OR Curtains) AND (Impregnated OR Impregnation OR Impregnating OR Incorporated OR Incorporation OR Incorporating OR Coated OR Coating OR Finish OR Finished OR Finishing OR Doped) AND (“Self-Disinfecting” OR Antimicrobial OR Antimicrobials OR Microbicide OR Microbicides OR Antibacterial OR Antibacterials OR Bactericide OR Bactericides OR Sporicide OR Sporicides OR Sporicidal OR Sporicidals OR Antifungical OR Antifungicals OR Antifungal OR Antifungals OR Fungicide OR Fungicides OR Antiviral OR Antivirals OR Viricide OR Viricides OR Biocide OR Biocides OR Inactivated OR Inactivating) AND (“Cross Infection” OR “Cross Infections” OR “Healthcare Associated Infection” OR “Healthcare Associated Infections” OR “Healthcare Acquired Infection” OR “Healthcare Acquired Infections” OR “Nosocomial Infection” OR “Nosocomial Infections” OR “Hospital Acquired Infection” OR “Hospital Acquired Infections” OR “Hospital Infection” OR “Hospital Infections” OR “HAI” OR “HAIs” OR Contamination OR Contaminations OR “Microbial Contamination” OR “Microbial Contaminations” OR “Microbial Load” OR “Microbial Loads” OR “Bacterial Contamination” OR “Bacterial Contaminations” OR “Bacterial Load” OR “Bacterial Loads” OR “Bacterial Count” OR “Bacterial Counts” OR Bioburden)	Article title, abstract, keywords	362

medRxiv	(Textile* OR Fabric* OR Woven* OR Linen* OR Bedding* OR Cloth* OR Uniform* OR Scrub* OR Gown* OR Curtain*) AND Antimicrobial*	Simple search	49
Google Scholar	Curtain* AND (Antimicrobial OR Antibacterial) AND (Contamination OR Infection)	Not applicable	---
	(Staff* OR Professional* OR Personnel OR Worker*) AND (Uniform* OR Cloth* OR Scrub* OR Gown*) AND (Impregnat*) AND (Antimicrobial OR Antibacterial)	Not applicable	---
	(Linen* OR Bedding* OR Cloth*) AND (Antimicrobial OR Antibacterial) AND (Contamination OR Infection)	Not applicable	---
	(Textile* OR Fabric* OR Woven* OR "Soft Surface") AND (Antimicrobial OR Antibacterial) AND (Contamination OR Infection)	Not applicable	---

SM2: Chart 2 - Reports excluded (by full-text reading) and their respective reasons for exclusion.

**Identification**	**Reasons for exclusion**
Ellingson et al., 2020	Reason 3
Elliott et al., 2019	Reason 1
Al-Tawfiq et al., 2019	Reason 2
Farid, 2019	Reason 1
Wilson, 2019	Reason 1
Madden, Heon, Sifri, 2018	Reason 1
Burke, Butler, 2018	Reason 5
Lautenbach et al., 2018	Reason 1
Rozman et al., 2017	Reason 1
Van Trang et al., 2017	Reason 1
Penes et al., 2017	Reason 3
Farid, Yeung, 2017	Reason 1
Anderson et al., 2016	Reason 1
Sifri, Enfield, Burke, 2016	Reason 1
Sifri, Burke, Enfield, 2016	Reason 5
Sridhar et al., 2016a	Reason 1
Sridhar et al., 2016b	Reason 1
Perelshtein, Perkas, Gedanken, 2016	Reason 1
Perelshtein et al., 2015	Reason 4
Perez et al., 2015	Reason 5
Tamimi, Carlino, Gerba, 2014	Reason 5

Boutin et al., 2013	Reason 1
Niiyama et al., 2013	Reason 7
Johnston, 2012	Reason 1
Schweizer et al., 2012	Reason 2
Di Nauta et al., 2011	Reason 1
Phillips, Taylor, Hastings, 2009	Reason 5
Taylor, Phillips, Hastings, 2009	Reason 5
Yoshino et al., 1998	Reason 2
Ayliffe, Beard, Filbey, 1962	Reason 5
Barnard, 1952	Reason 6

Caption: Reason 1: Material on the theme published, such as editorial, letter to the editor, book, book chapter, thesis, dissertation or abstract presented in a scientific event; Reason 2: The study does not specify the antimicrobial substance impregnated in the textiles; Reason 3: The study does not specify applicability of the antimicrobial textiles in the health service; Reason 4: The study does not assess microbial load in the antimicrobial textiles and/or the HAI rates by means of a theoretical framework or indicators; Reason 5: In addition to the intervention of interest (antimicrobial textiles), the study addresses concomitant use of other antimicrobial surfaces in the health service; Reason 6: The study does not present sufficient information/data for characterization and analysis of methodological quality/risk of bias; and Reason 7: The study investigates a given non-textile antimicrobial material fixed to conventional textiles (non-antimicrobial).

REFERENCES

Anderson DJ, Warren B, Addison R, Sharma-Kuinkel BK, Lokhnygina Y, Rojas LJ, et al. Molecular Analysis of Transmission Events in the Antimicrobial Scrub and Transmission (ASCOT) Trial. In: Oral Abstracts of the Infectious Diseases Week 2016; 2016; New Orleans. Open Forum Infect Dis 2016;3(supp.1):943. DOI: 10.1093/ofid/ofw194.83

Al-Tawfiq JA, Bazzi AM, Rabaan AA, Okeahialam C. The effectiveness of antibacterial curtains in comparison with standard privacy curtains against transmission of microorganisms in a hospital setting. Infez Med 2019;27(2):149-54.

Ayliffe GAJ, Beard MA, Filbey J. A system of air recirculation and antibacterial surface treatment in a surgical ward. J Clin Pathol 1962;15(3):242-6. DOI: 10.1136/jcp.15.3.242

Barnard HF. The sterilization of woollen fabrics. Br Med J 1952;1(4748):21-4. DOI: 10.1136/bmj.1.4748.21

Boutin M, Thom K, Zhan M, Johnson JK. A novel agent to decrease contamination on hospital scrubs. In: Poster Abstracts of the Infectious Diseases Week 2013; 2013; San Francisco. Poster Abstract:1694.

Burke GH, Butler JP. Analysis of the role of copper impregnated composite hard surfaces, bed linens and patient gowns in reducing healthcare-associated infection rates. Int J Infect Control 2018;14(1). DOI: 10.3396/ijic.v14i1.18002

Di Nauta A, Baldan R, Romanò C, Drago L, Elia F, Cirillo D. Antimicrobial effect of treated medical textiles. In: Posters of the 21st European Congress of Clinical Microbiology and Infectious Diseases / 27th International Congress of Chemotherapy; 2011; Milan. Clin Microbiol Infect 2011;17(supp.4):P1159.

Elliott SP, Ellingson K, Pogebra-Brown K, Gerba CP. A Novel Antimicrobial Surface Coating Demonstrates Persistent Reduction of both Microbial Burden and Healthcare-Associated Infections at Two High-acuity Hospitals. In: Poster Abstracts of the Infectious Diseases Week 2019; 2019; Washington DC. Open Forum Infect Dis 2019;6(supp.2):1216. DOI: 10.1093/ofid/ofz360.1079

Ellingson KD, Pogreba-Brown K, Gerba CP, Elliott SP. Impact of a Novel Antimicrobial Surface Coating on Health Care-Associated Infections and Environmental Bioburden at 2 Urban Hospitals. Clin Infect Dis 2020;71(8):1807-13. DOI: 10.1093/cid/ciz1077

Farid A, Yeung KL. A multi-level antimicrobial coating for patient privacy curtains: implication of a new system to combat hospital acquired infections (HAIs). In: Meeting abstracts from the 4th International Conference on Prevention & Infection Control; 2017; Geneva. Antimicrob Resist Infect Control 2017;6(supp.3):P391.

Farid A. Safe antimicrobial systems for infection control in healthcare settings [thesis (Ph.D.)]. China: Hong Kong University of Science and Technology; 2019. DOI: 10.14711/thesis-991012753760003412

Johnston S. The effect of antimicrobial impregnated fabrics on the contamination of healthcare workers uniforms in clinical environments [thesis (Ph.D.)]. Northern Ireland: University of Ulster; 2012.

Lautenbach E, Pegues D, Fuchs B, Martin N, Nachamkin I, Bilker W, et al. A randomized controlled trial of the effect of accelerated copper textiles on healthcare-associated infections and multidrug-resistant organisms: The “Investigating Microbial Pathogen Activity of Copper Textiles” (IMPACT) study. In: Poster Abstracts of the Infectious Diseases Week 2018; 2018; San Francisco. Open Forum Infect Dis 2018;5(supp.1):1961. DOI: 10.1093/ofid/ofy210.1617

Madden GR, Heon BE, Sifri CD. Multi-Drug Resistant Organism Acquisition and Clostridium difficile Infection with Copper-Impregnated Linens. In: Abstracts of The Society for Healthcare Epidemiology of America Spring 2018 Conference; 2018; Portland. Poster:201.

Niiyama N, Sasahara T, Mase H, Abe M, Saito H, Katsuoka K. Use of Copper Alloy for Preventing Transmission of Methicillin-resistant Staphylococcus aureus Contamination in the Dermatology Ward. Acta Derm Venereol 2013;93(3):294-300. DOI: 10.2340/00015555-1472

Penes ON, Neagu AM, Plata F, Paun SD. Fabric with silver thread role in the control of bacterial contamination in critically ill patients. Ind Text 2017;68(1):54-7. DOI: 10.35530/IT.068.01.1341

Perelshtein I, Lipovsky A, Perkas N, Tzanov T, Arguirova M, Leseva M, et al. Making the hospital a safer place by sonochemical coating of all its textiles with antibacterial nanoparticles. Ultrason Sonochem 2015;25:82-8. DOI: 10.1016/j.ultsonch.2014.12.012

Perelshtein I, Perkas N, Gedanken A. Making the hospital a safer place by the sonochemical coating of textiles by antibacterial nanoparticles. In: Grumezescu AM, editor. Surface Chemistry of Nanobiomaterials. Applications of Nanobiomaterials. William Andrew Publishing; 2016. p.71-105. DOI: 10.1016/B978-0-323-42861-3.00003-0

Perez V, Mena KD, Watson HN, Prater RB, McIntyre JL. Evaluation and quantitative microbial risk assessment of a unique antimicrobial agent for hospital surface treatment. Am J Infect Control 2015;43(11):1201-7. DOI: 10.1016/j.ajic.2015.06.013

Phillips P, Taylor L, Hastings R. Silver ion antimicrobial technology: decontamination in a nursing home. Br J Community Nurs 2009;14(Sup3):S25-9. DOI: 10.12968/bjcn.2009.14.Sup3.85161

Rozman U, Pavlinić DZ, Pal E, Gönc V, Turk SS. Efficiency of Medical Workers' Uniforms with Antimicrobial Activity. In: Kumar B, Thakur S, editors. Textiles for Advanced Applications. IntechOpen; 2017. p. 255-73. DOI: 10.5772/intechopen.68830

Schweizer M, Graham M, Ohl M, Heilmann K, Boyken L, Diekema D. Novel hospital curtains with antimicrobial properties: a randomized, controlled trial. Infect Control Hosp Epidemiol 2012;33(11):1081-5. DOI: 10.1086/668022

Sifri CD, Burke GH, Enfield KB. Reduced health care-associated infections in an acute care community hospital using a combination of self-disinfecting copper-impregnated composite hard surfaces and linens. Am J Infect Control 2016;44(12):1565-71. DOI: 10.1016/j.ajic.2016.07.007

Sifri CD, Enfield K, Burke G. Reduced Healthcare-Associated Infections in an Acute Care Community Hospital Using a Combination of Self-Disinfecting Copper-Impregnated Composite Hard Surfaces and Linens. In: Poster Abstracts of the Infectious Diseases Week 2016; 2016; New Orleans. Open Forum Infect Dis 2016;3(supp.1):263. DOI: 10.1093/ofid/ofw172.129

Sridhar SA, Ledeboer NA, Nanchal RS, Mackey T, Graham MB, VanDerSlik A, et al. Antimicrobial Curtains: Are They as Clean as You Think? Infect Control Hosp Epidemiol 2016a;37(10):1260-2. DOI: 10.1017/ice.2016.186

Sridhar S, Ledeboer N, Nanchal R, Mackey T, Graham MB, Vanderslik A, et al. Are Antimicrobial Curtains as Clean as You Think? In: Poster Abstracts of the Infectious Diseases Week 2016; 2016; New Orleans. Open Forum Infect Dis 2016b;3(supp.1):260. DOI: 10.1093/ofid/ofw172.126

Tamimi AH, Carlino S, Gerba CP. Long-term efficacy of a self-disinfecting coating in an intensive care unit. Am J Infect Control 2014;42(11):1178-81. DOI: 10.1016/j.ajic.2014.07.005

Taylor L, Phillips P, Hastings R. Reduction of bacterial contamination in a healthcare environment by silver antimicrobial technology. J Infect Prev 2009;10(1):6-12. DOI: 10.1177/1757177408099083

Van Trang DT, Thu LTA, Nga TTT, Kurosu H. Effectiveness of antibacterial linen in prevention of hospital acquired infections. In: Abstracts of the 30th International Congress of Chemotherapy and Infection; 2017; Taipei. Int J Antimicrob Agents 2017;50(supp.2):OS9-4.

Wilson GM. Environmental and pharmaceutical risk factors for the transmission of Clostridium difficile and other multi-drug resistant hospital acquired infections [Dissertation (Ph.D.)]. United States of America: University of Iowa; 2019. DOI: 10.17077/etd.cp4w-j0zs

Yoshino S, Fujita N, Nakamura Y, Komori T, Takabayashi T, Yoshimura M. A Clinical Evaluation of Antibacterial Linens on Nosocomial MRSA Infection in An Intensive Care Unit. Environm Infect 1998;13(2):113-7. DOI: 10.11550/jsei1986.13.113
